# 2D mental whiteboards: Multidimensional spatial coding of serial order in verbal working memory

**DOI:** 10.1007/s00426-026-02267-9

**Published:** 2026-05-22

**Authors:** Elger Abrahamse, Jean-Philippe van Dijck, Matthias Hartmann

**Affiliations:** 1 Tilburg University, Warandelaan 2, Tilburg, P.O. Box 90153, 5000 LE The Netherlands; 2 Universidad del Atlántico Medio, Las Palmas de Gran Canaria, Spain; 3 Thomas More University of Applied Sciences, Antwerp, Belgium; 4 Ghent University, Ghent, Belgium; 5 UniDistance Suisse, Brig, Switzerland

## Abstract

Previous work has shown that serial order in verbal working memory triggers the spontaneous formation of a mental line on which the sequence of items is coded. Recent studies show that the orientation (horizontal versus vertical) and coding direction (e.g., left-to right versus right-to-left) of this spatialization can flexibly be adapted to match the task context. Here we investigate whether people spatialize a sequence of verbal items using multiple spatial dimensions simultaneously if the task probes such strategy. In two experiments, participants had to encode a letter sequence and maintain it for later reproduction. In between, they performed a vowel-consonant classification task on items that were part of the memorized sequence to probe for spatialization. Classification occurred via horizontal or vertical saccade responses that varied unpredictably from trial to trial, with a fixed (Experiment 1) or variable (Experiment 2) stimulus-response mapping. Spatial biases were observed for both horizontally (early-left, late-right) and vertically (early-up, late-down) oriented classification trials (mixed within the same block), indicating that the item sequence was spatialized in a multidimensional format (i.e. exploiting both the vertical and horizontal axes). The horizontal and vertical biases were also correlated with each other. Overall, we show for the first time that task context can probe a strategy in which people spatially code a sequence of verbal items using multiple spatial dimensions simultaneously.

‘‘Things that in the physical-behavioral world do not have a spatial quality, are made to have such in consciousness. Otherwise we cannot be conscious of them. This we shall call spatialization”. Julian Jaynes (1976).

## Introduction

The ability to temporarily maintain unfamiliar sequences of verbal and/or auditory items is at the core of verbal working memory. It contributes to broader cognitive functions such as language use, planning, problem solving, and decision-making (e.g. Baddeley, 2003). Traditionally, serial order ability has been attributed to a phonological loop, in which unfamiliar sequences (e.g., lists of digits, letters, or words) are encoded in phonological form and actively maintained through articulatory rehearsal (e.g., Baddeley, 1986). Whereas the phonological loop remains a core element in recent work on verbal serial order, additional mechanisms have been proposed more recently to complement the loop. Specifically, it has been proposed that, besides the phonological loop, attention-based mechanisms are at play in serial order ability (e.g. Abrahamse et al., 2017; Camos, 2015).

A concrete attention-based mechanism has been proposed in the mental whiteboard hypothesis (e.g. Abrahamse et al., 2014, 2017). Inspired by empirical observations in the domain of numerical cognition (e.g. Abrahamse et al., 2016; van Dijck & Fias, 2011; Van Dijck et al., 2014), it was postulated that verbal working memory exploits (internal) spatial coding as a tool to support serial order (e.g. Van Dijck et al., 2013). Specifically, the mental whiteboard hypothesis holds that, when we are challenged to maintain an unfamiliar series of verbal items, encoding involves the spontaneous^1^ generation of a spatial template in mind – metaphorically coined the ‘mental whiteboard’. The serial organization of the items in a sequence can then be encoded and maintained by binding each item to systematically aligned coordinates (e.g. from left to right) of the spatial template (e.g., Abrahamse et al., 2014, 2017; Van Dijck et al., 2013; van Dijck & Fias, 2011), as if people ‘write down’ the items on the mental whiteboard. Once a spatially defined serial order representation is installed, internal spatial attention can be employed to search through the item series, and select specific items for retrieval and further operations (e.g. Abrahamse et al., 2017; Rinaldi et al., 2015 Sahan et al., 2022). The latter selection of a target item on the spatially defined mental whiteboard underlies the spatial biases observed in studies supporting the hypothesis (see below). Overall, the mental whiteboard hypothesis assumes that spatial coding is a functional tool in the toolkit that is verbal working memory, building on a (latent) potential to exploit spatial coding to support formally non-spatial tasks (e.g. Bottini & Doeller, 2020; Van Dijck et al., 2020) that is then functionally shaped by reading and writing experience (e.g. Guida et al., 2018).

The mental whiteboard hypothesis was inspired by the finding that a systematic left-to-right bias accompanies the retrieval of items from verbal working memory across participants from Western cultures (e.g. van Dijck & Fias, 2011; Van Dijck et al., 2013). Specifically, retrieval of an item early in a (formally) non-spatial^2^ sequence that is maintained in working memory, results in a relative left-ward bias in information processing, whereas retrieval of later items increasingly shifts this bias more to the right. This so-called Spatial Positional Association of Response Codes (SPoARC; Guida & Lavielle-Guida, 2014) or Ordinal Position (OP) effect (Ginsburg et al., 2014) occurs *spontaneously*, even when task instructions and stimuli do not signal any formal need for spatial processing (e.g. Sahan et al., 2022; Schroth et al., 2025). The SPoARC effect has by now been reported across ample studies (for reviews see Abrahamse et al., 2017; Ftaïta et al., 2023), using various types of items (e.g., letters, Arabic digits, words, verbalizable images) and responses (e.g., key-presses, eye movements), indicating its robust and generalizable nature (e.g., Bottini et al. 2016; Ginsburg et al. 2017; Guida and Porret 2022; Shi et al. 2020a, b; Wang et al. 2019; Hartmann et al. 2021; Rinaldi et al. 2015; Van Dijck et al. 2014; Van Dijck et al., 2013; van Dijck & Fias, 2011; Sahan et al., 2022). Further support for the mental whiteboard hypothesis comes from studies showing that serial order in verbal working memory features a right-to-left orientation (i.e. a reversed SPoARC effect) in Arabic populations that read and write from the right to the left (e.g., Guida et al., 2018; Rasoulzadeh et al., 2024), and that no systematic spatial orientation is observed for illiterates (Guida et al., 2018) or early blind people that were never exposed to visuo-spatial reading and writing processes (Bottini et al., 2016). These observations fit the notion that reading and writing experience shape the horizontal orientation of serial order coding in verbal working memory.

Besides the robust SPoARC effects observed in previous studies, support for the mental whiteboard hypothesis is triangulated by neuroscientific and computational work. With respect to the former, studies have demonstrated that brain areas (e.g., hippocampus and intraparietal sulcus; Attout et al., 2019; Cristoforetti et al., 2022; Zhou et al., 2021) and neural processing signatures (e.g., Rasoulzadeh et al., 2021) that are traditionally linked to external spatial processing, are also involved in serial order retrieval. Additionally, the mental whiteboard hypothesis maps naturally onto the notion of position marking that is central to the main computational models of serial order (cf. Hurlstone et al., 2014; see also Abrahamse et al., 2014). Hence, potentially, coordinates on the spatially defined mental whiteboard are a concrete implementation of the otherwise abstract notion of position markers.

### Spatial coding as a functional tool in verbal working memory

Despite the ample empirical support for, and the theoretical reach of, the mental whiteboard hypothesis outlined above, one pillar of the hypothesis has remained untested directly: Is spatial coding indeed *functionally* involved in serial order ability, as opposed to merely being an (intriguing but) causally unrelated epiphenomenon? A definite answer to this question would require a clear association between the spontaneous use of spatial coding and serial order performance. Yet, it is theoretically challenging to quantify the use of spatial coding (e.g. the numerical size of the SPoARC effect may not linearly map onto the systematicity with which spatial coding is exploited for a given individual), and serial order performance can be solid also in the absence of spatial coding (e.g. by falling back onto alternative order mechanisms such as the phonological loop). At the same time, dedicated training studies are lacking that test if and how serial order ability *in working memory* can benefit from instructed and/or trained use of spatialization.

Nevertheless, indirect support for a functional use of spatial coding in serial order working memory exists. First, *externally* induced spatial structure has been shown to improve serial order performance (e.g., Darling et al., 2017; Yousif et al., 2021), fitting the notion that *spontaneous* generation of spatial structure, too, would be a performance-enhancing policy. Second, the SPoARC effect correlates both with verbal working memory span (e.g., Van Dijck et al., 2022; Tian & Fischer-Baum, 2025; Vivion et al., 2025) and with math performance (e.g., Van Dijck et al., 2022). Hence, spatial coding has been associated with enhanced performance on tasks that are known to build on serial order working memory. Third, Tian and Fischer-Baum (2025) showed that the SPoARC effect was associated with higher serial order capacity, while it was not associated with mere item-level retrieval performance. This indicates that spatial coding is specifically linked to serial order processing.

Finally, the mental whiteboard is supported by the principle of parsimony. If spatial coding is not functionally involved in serial order ability, the SPoARC effects requires an alternative explanation. Several alternative accounts hold such potential of explaining away SPoARC effects as an epiphenomenon with respect to serial order performance in working memory. Specifically, according to the polarity coding principle (Proctor & Cho, 2006), conceptual metaphor theory (Lakoff & Johnson, 2003; Winter et al., 2015), and/or indirect spatial-numerical association logic (Botvinick & Watanabe, 2007; Marshuetz, 2005), the SPoARC effect may result from linguistic and/or response-based mechanisms that intrinsically have little to do with serial order ability in verbal working memory (for elaboration, see Hartmann et al., 2021). Yet, these alternatives run into problems with respect to vertically oriented spatial coding that has recently been observed by Hartmann et al. (2021), in which serial order mapping was observed to be oriented from top (early items) to bottom (later items).^3^ Whereas this outcome is more or less predicted (*a posteriori*) by the mental whiteboard logic (because reading and writing directions are thought to shape spatial coding for serial order, and we typically read from top to bottom; Abrahamse et al., 2017; Guida et al., 2018), polarity coding, metaphor theory, and indirect spatial-numerical association logic would each predict the opposite direction (bottom-to-top mapping).^4^ Moreover, in the study by Hartmann et al. (2021), horizontal and vertical SPoARC effects were positively correlated with each other, which suggests that both build on the same mechanism. As far as we are aware, there is no account besides the mental whiteboard hypothesis that can explain this correlated left-to-right and top-to-bottom oriented spatial coding in Western populations. Overall, we argue that solid support exists *against* the notion that spatial biases observed in serial order working memory are mere epiphenomena.

### Flexible use of the mental whiteboard

If the mental whiteboard is indeed a functional tool of verbal working memory, spatial coding on the whiteboard should be under goal-directed control. That is, its exploitation should be flexibly calibrated to the task context in order to optimally support serial order processing. Several studies suggest such flexibility in the use of the mental whiteboard in function of task demands. For example, Guida et al. (2020) varied the orientation with which to-be-maintained items were presented during encoding (i.e., central, left-to-right on the screen, or right-to-left on the screen). They observed similar-sized SPoARC effect across all conditions – but with an inverted effect in the right-to-left condition. Hence, the filling out of the mental whiteboard was swiftly adjusted to item presentation.

As another example, let us zoom in more deeply on the study by Hartmann et al. (2021) already mentioned above, as the current study directly builds on their design and findings. They had participants first encode and maintain a four-letter sequence consisting of two vowels and two consonants (*phase 1* of a trial block). In a later *phase 3* of the trial block, participants were asked to speak aloud the memorized sequence in order to verify correct recall of the sequence. In between these encoding and recall phases, *phase 2* consisted of a series of go-nogo trials in which participants were asked to classify a target letter presented in the center of the screen as either a vowel or consonant as quickly and correctly as possible – but *only* for target letters that were part of the memorized sequence (i.e. go trials). No response was required for target letters outside the memorized sequence (i.e. nogo trials), in order to force participants to scan the sequence maintained in verbal working memory just before a response was given (on go trials). This is all part of the typical design for studying the SPoARC effect (cf. the seminal paper by Van Dijck et al., 2011).

In order to capture the potentially spatialized format of sequences maintained in verbal working memory, typical SPoARC studies employ spatially outlined response options. An interesting aspect of the study by Hartmann et al. (2021) concerned the use of saccadic responses (rather than key-press responses used in most previous studies): Every time a target letter appeared on screen in phase 2 for classification, two saccade trigger response boxes simultaneously appeared to the left and right of the target letter, and participants had to move their eyes to the box corresponding to the classification response (participants had learned before which box was for vowel-responses and which was for consonant-responses). With this design, they were able to replicate the horizontal SPoARC effect with saccadic responses.

Additionally, employing saccadic responses enabled Hartmann et al. (2021) to also test for a vertically oriented SPoARC effect by adding trial blocks in which the saccade trigger response boxes were not presented to the left or right of the target letter position, but rather above and below it. By aligning the saccadic response boxes vertically on the screen, they demonstrated a reliable vertical SPoARC effect that was oriented from top (early items) to bottom (later items), and that correlated with the horizontal SPoARC effect. Since in their design participants always performed all trial blocks of one spatial orientation in one session (e.g., starting with only horizontal blocks) before moving to another session in which only the alternative spatial orientation (e.g. vertical blocks) was performed, the optimal spatial format for encoding could be readily predicted (i.e. participants had knowledge about the orientation of trials in the upcoming block). Hence, participants could exploit instructions and/or initial experience with the task to proactively determine the use of either a horizontally or vertically oriented spatial representation for serial order encoding – matching the orientation of the saccade response boxes.

Such context-sensitivity is a hallmark feature of goal-directed mechanisms (e.g., Abrahamse et al., 2016), and aligns with the mental whiteboard hypothesis in terms of the proactive installment of task-appropriate spatial representations.

### The current study: Context-dependent coding on the multidimensional mental whiteboard

Whereas previous work shows flexible, context-dependent coding of order on the mental whiteboard (e.g. Guida et al., 2020; Hartmann et al., 2021), in these studies people could suffice with serial order coding along a single, unidimensional mental line – with flexibility relating to either the context-dependent orientation of the mental line (Hartmann et al., 2021) or the context-dependent mapping of items on coordinates along the line (Guida et al., 2020). But the mental whiteboard promises to be a goal-adaptive, *multidimensional* workspace, theoretically affording higher levels of flexibility than the mere use of unidimensional mental lines. When confronted with more complex task contexts that require task operations across different orientations *concurrently*, can people use multiple (i.e. combined horizontal and vertical) spatial axes to support serial order ability in a context-dependent fashion?

To test this, here we push the need for flexible coding of serial order beyond what was done in the previous studies by Hartmann et al. (2021). Specifically, we adjusted their design by varying the response configuration (i.e. horizontally versus vertically outlined saccade responses boxes) from trial to trial within phase 2 of the task (see above), rather than between sessions as in their study. Rather than entertaining a single order representation that matches the orientation of all the trials within a block (as was possible in the design by Hartmann et al., 2021), here participants are required to exploit both the horizontal and vertical axis for serial order coding – if they are able and willing to optimally fit the order representation to the task context.

Several outcome scenarios can be anticipated (see Table 1 for an overview), each with different theoretical implications. The **multidimensional scenario** holds that people can adjust to the trial-by-trial uncertainty in the orientation of response mapping by moving beyond the use of unidimensional mental lines (cf. Hartmann et al., 2021) and exploit the enhanced flexibility that the notion of a two-dimensional workspace such as the mental whiteboard affords. We can think of at least two possible strategies for context-specific order representations to fit such a multidimensional context, with each strategy probed either by the task instructions or by initial on-task experience. First, participants may proactively encode the sequence along a single, diagonally oriented mental line that can be used for both horizontal and vertical trials in the classification task. Second, participants may proactively encode and maintain not only one (as indicated by previous work) but multiple distinct mental lines for serial order – a horizontally oriented representation to use for horizontal trials, and a vertically oriented representation to use on vertical trials. In order to exploit these multiple representations in a context-appropriate manner, participants then flexibly adjust the orientation of the currently operational representation *on the fly* depending on current trial context (i.e. reactive control that activates in working memory the order representation that best aligns with the saccade trigger response boxes on each trial). In each of these strategies, the horizontal and vertical axes are simultaneously exploited to support serial order ability – in line with multidimensional coding. Overall, independently of the precise strategy that underlies it, the multidimensional scenario would predict (a) that both a horizontal and vertical SPoARC effect are jointly observed at the group level, and (b) that these SPoARC effects are positively correlated with each other, because individuals that are prone to spatialize serial order would show the SPoARC effect across both horizontal and vertical trials more strongly and systematically than individuals lacking this proneness.

**Table 1:** Predictions of effects in function of the different scenario’s outlined in the main text

Account/Scenario	H- SPoARC	V- SPoARC	COR
*Epiphenomenal accounts*: polarity coding, metaphor theory, and indirect spatial-numerical association logic	**L-R**	**B-T**	**?**
*Mental Whiteboard hypothesis*:			
No-spatialization scenario	**X**	**X**	**X**
Unidimensional-homogeneous scenario	**L-R**	**X**	**X**
Unidimensional-heterogeneous scenario	**L-R**	**T-B**	**-**
Multidimensional scenario	**L-R**	**T-B**	**+**
- Diagonal coding			
- Multiple mental lines			

*H-SPoARC* horizontal SPoARC effec, *V-SPoARC* vertical SPoARC effect, *COR* correlation between the horizontal and vertical SPoARC effects (with + = positive correlation, - = negative correlation, and X = no correlation); L-R = left-to-right mapping; B-T = bottom-to-top mapping; T-B = top-to-bottom mapping

However, it is also possible that participants are not able (or willing) to go beyond serial order coding along an unidimensional mental line. In the **unidimensional-homogenous scenario**, participants proactively select a single spatial orientation to dominate spatial coding of order (i.e. either a horizontally or vertically oriented representation), accepting the implication that the order representation mismatches the response box configuration on half of the trials (e.g. an horizontally oriented order representation would mismatch the response box configuration on all vertical trials). If the majority of the participants select the same orientation – hence, when there exists relative homogeneity *across* participants – this would predict a reliable SPoARC effect to be observed for either only the horizontal trials or only the vertical trials. Indeed, given that Woodin and Winter (2018) showed that the vast majority (79%) of participants use the horizontal dimension when asked to freely place a sequence of four numbers in space, we specifically predict a horizontally (but not vertically) oriented SPoARC effect because the horizontal dimension is more intuitive than the vertical for short item sequences. Finally, the homogenous-dominance scenario would not predict a correlation between horizontal and vertical SPoARC effects because only a single orientation is exploited.

A third possibility is here coined the **unidimensional-heterogenous scenario**, holding that a single spatial orientation is proactively selected to dominate spatial coding of order for each individual – but that individuals exhibit strong individual differences in adopting either a horizontally or a vertically oriented order representation. This would predict reliable SPoARC effects for both the horizontally and vertically oriented trial types, as well as a negative correlation between these effects at the group level (because an individual coding a sequence from left-to-right would not use vertical coding, and vice versa).

Finally, no reliable SPoARC effects may be observed at all, neither for the horizontally nor for the vertical oriented trials (i.e., the **no-spatialization scenario**). This outcome would be expected if the unpredictability of spatial response box orientations renders it less functional (or even dysfunctional) to support serial order processing with spatialized order representations, and individuals fall back entirely on their phonological loop for non-spatial order maintenance. Hence, this outcome would suggest that the unpredictable task context probes participants to not invest effort into the proactive installment of any spatially defined order representation.

Overall, the current study has two main purposes. First, we aimed to replicate the core findings of Hartmann et al. (2021) – that is, a top-to-bottom oriented vertical SPoARC effect that correlates with the horizontal SPoARC effect – because of their relevance for the discussion about the functional involvement of spontaneous spatial coding in verbal working memory. Support for accounts that posit spatial coding as an epiphenomenon in serial order performance would predict a bottom-to-top orientation of the SPoARC effect in vertical trials, whereas the mental whiteboard hypothesis would predict a top-to-bottom orientation (see above). The second aim is to explore the potential of multidimensional coding. A functional role of spatial coding in verbal working memory that only exploits simple mental lines would be supported by outcomes under the unidimensional-homogenous or unidimensional-heterogenous scenarios, whereas only outcomes under the multidimensional scenario would support people’s ability to exploit the full functionality that a 2D mental whiteboard affords. Indeed, such advanced flexibility to adjust to multidimensional task demands would further reinforce a functional account of spatial coding as a goal-directed tool in serial order ability.

#### Experiment 1

Following the study by Hartmann et al. (2021), in Experiment 1, after memorizing a sequence of four letters (phase 1) that later was verified for correct recall (phase 3), participants performed a vowel-consonant classification task in phase 2 (only for target letters part of the memorized sequence, in line with the typical go-nogo instructions). For each trial in phase 2, saccade response boxes unpredictably appeared either above and below the target letter, or to the left and right of it. This way, the participant was only informed about the orientation of the response boxes upon target presentation, eliminating the possibility to proactively adjust the orientation for the entire block via prior knowledge about the type of trials being encountered in phase 2. In order to facilitate the task, the vowel-response and consonant-response boxes were always in the same location for each orientation (counterbalanced over participants). So, for example, for one participant, the vowel-response box was always to the left (for horizontal trials) and top (for vertical trials), while the consonant-response box was always to the right and bottom. This design allows us to test for the first time if an individual will show both horizontal and vertical SPoARC effects in the same block, and if so, if these are correlated with each other.

## Methods

### Participants

Thirty-four undergraduate students participated in Experiment 1 in return for course credit (mean age was 21.8 years, ranging from 18 to 39; 27 female). The sample size of *n* = 34 was defined by an a-priori power analysis using G*Power 3 (Faul et al., 2007) based on a one-sample *t*-test (i.e., following the conventional approach to quantify the strength of the SPoARC effect by testing the individual regression weights against zero; see data analysis), with a probability greater than 0.8 for detecting an effect size of *d* ≥ 0.5, assuming a two-sided criterion for detection that allows for a maximum Type I error of α = 0.05. Such a sample size is in the same range as in previous studies on the SPoARC effect, and has been shown to be large enough to also detect correlations between horizontal and vertical associations (e.g., Hartmann et al., 2021; Van Dijck et al., 2014). Participants gave informed consent prior to the study, and the study protocol was approved by the Ethics Committee of UniDistance Suisse. All participants reported German as their native language.

### Apparatus and experimental setting

We used an EyeLink 1000 eye tracker (SR Research Ltd., Ontarion, Canada) with a sampling rate of 1000 Hz. Eye-tracking data was parsed into saccades using the manufacturer’s default parameters. Participants placed their heads on a chin- and forehead rest. The head-to-screen distance was 800 mm. Stimuli were presented on a 520 × 300 mm monitor (1920 × 1080 px; 36° × 21.24°).

### Stimuli

Stimuli were identical to Hartmann et al. (2021). Specifically, we used the four vowels “a”, “e”, “i”, “u” and the four consonants “c”, “n”, “r”, “s” for creating the to-be-memorized four-letter-sequences as well as for the target letters in the consonant-vowel-classification task. To generate four-letter sequences, we began by creating every possible combination of two vowels and two consonants. Out of these, eighteen combinations were randomly selected. For each of these 18 distinct combinations, the four letters were arranged twice in a pseudo-random order, resulting in 36 unique sequences that were used for all participants. The arrangement of the sequences ensured that each serial position (1, 2, 3, 4) was equally occupied by vowels and consonants across all sequences. Additionally, we made sure that all six possible vowel-consonant configurations (e.g., consonant-vowel-consonant-vowel) were represented equally. One extra sequence was randomly selected to be used for a practice block (see below).

### Procedure

Participants were positioned in front of the eye-tracker. After completing the 9-point calibration procedure, task instructions were displayed on the screen. Participants were informed that the task consisted of several blocks of the three phases detailed below. They were told to respond by making either leftward and rightward, or upward and downward eye movements – depending on the location of saccade response boxes presented on each specific trial. Participants then performed two practice tasks. The first practice task involved a consonant-vowel classification task. Each of the eight letters was presented three times, resulting in 24 practice trials (12 horizontal and 12 vertical responses), designed to familiarize participants with the saccade response setting. The second practice task mirrored the main experimental procedure and included the three phases of (1) memorizing the sequence, (2) consonant-vowel classification task, and (3) verification of the sequence, as established for the SPoARC effect (cf. Ginsburg et al., 2017; van Dijck & Fias, 2011). In the first phase of memorizing a sequence, participants were asked to memorize a four-letter sequence that needed to be recalled later (phase 3) in the correct order. The four letters of the sequence were displayed sequentially in the center of the screen (26 pt. Arial), each for 1000 ms, separated by 10 ms blank screens. After viewing the four letters, participants decided if they wanted to see the sequence again (pressing “j” for “yes”), or move to the next phase (pressing “n” for “no”). They could repeat the sequence as many times as needed.

In the second phase, participants performed a consonant-vowel classification task, requiring them to quickly and accurately classify centrally presented target letters as either a vowel or a consonant. There were 16 trials in each of the classification blocks of phase 2 (1 practice block and 36 experimental blocks). During each block, each of the eight letters from the entire stimulus set was shown twice in the center of the screen (26 pt. Arial) in a random order, once requiring a horizontal response and once a vertical response. Specifically, the target letter stimuli were accompanied by two saccade trigger response boxes (100 × 100 px; ~ 2.0 × 2.0°), positioned either to the left and right (horizontal dimension) or at the top and bottom (vertical dimension) of the screen. The boxes were 440 px (~ 8.6°) from the center of the screen. One box displayed a capital “V” for vowel (Vokal in German), and the other a capital “K” for consonant (Konsonant in German), both also in 26 pt. Arial.

**Fig. 1:**
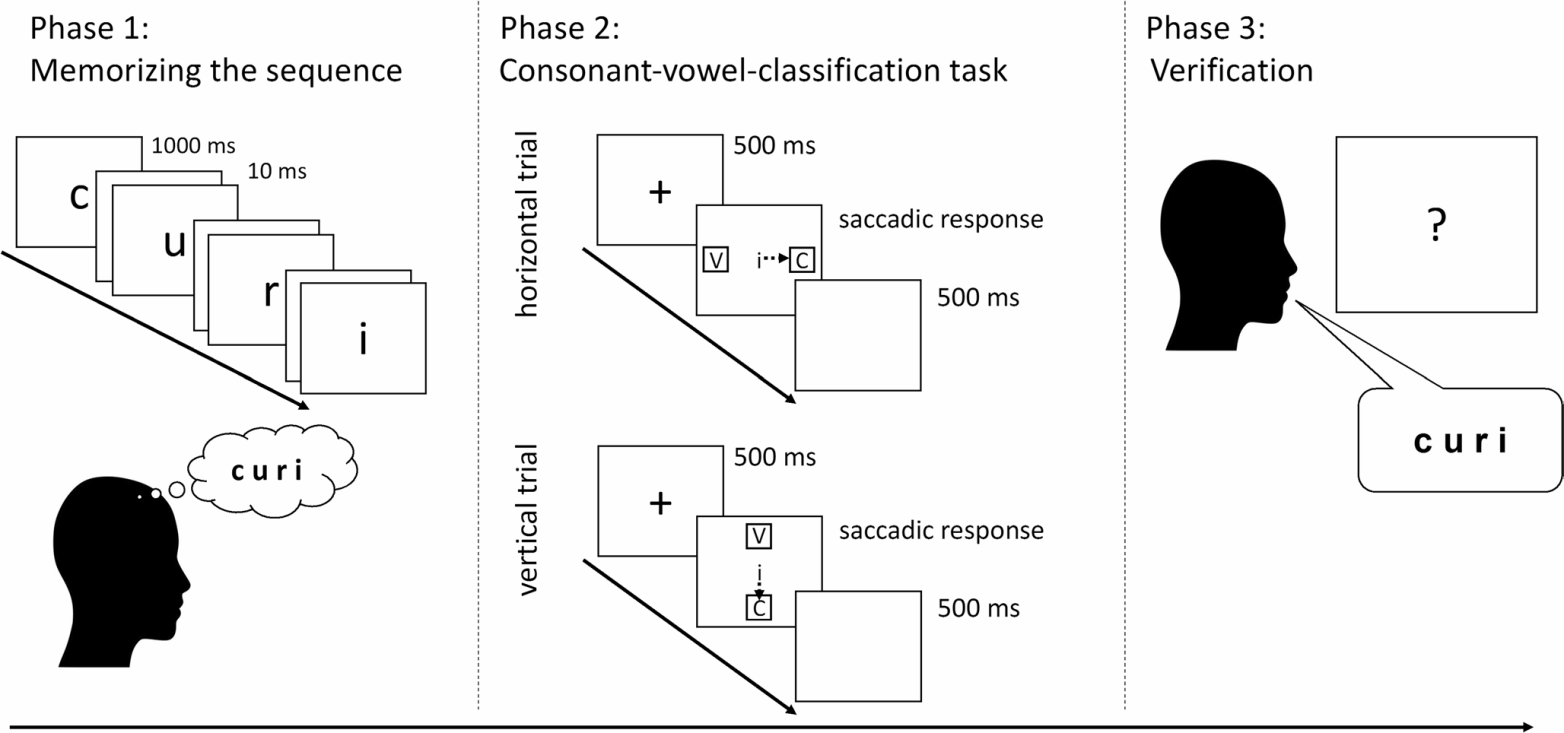
Experimental procedure of Experiment 1. Phase 2 shows examples of a horizontal and vertical go-trial. In case of a nogo-trial, participants were instructed to remain with their eyes fixated on the centre during 3000 ms. The three-phase procedure was repeated 36 times (plus additional runs for incorrectly verified sequences), each with 16 phase 2 trials

For half of participants, vowels required a left and up response, and consonants a right and down response, and this mapping was reversed for the other half of participants (i.e. the mapping remained the same across the experiment for each participant). Before starting the classification task, a fixation dot was displayed in the center of the screen to serve as a drift correction for the eye-tracker. Within the classification task, each trial started with a fixation cross presented at the center of the screen for 500ms, followed by the target letter (see Fig. 1). Crucially, participants were instructed to respond only to the letters that were part of the memorized sequence (8 go-trials per classification block). This requirement ensured that the memorized sequence remained in working memory during the classification task, and was activated on each trial for go versus nogo differentiation. For these go-trials, participants classified the target letter by looking at the corresponding “V” or “K” box. For letters not in the memorized sequence (8 nogo-trials per classification task), participants were to keep their eyes fixed at the central letter, and the next trial began automatically after 3000 ms. The word “Falsch” (“wrong”) appeared in red in the center of the screen if participants (1) looked at the wrong box during a go-trial, (2) did not look at a box within 3000 ms during a go-trial, or (3) looked at a box during a no-go-trial. A 500 ms blank screen was shown before the next trial began.

In the final phase (phase 3), participants were asked to verbally recall the previously memorized sequence (in order of phase 1 presentation) in order to verify correct maintenance of the sequence. A question mark was displayed in the center of the screen after the classification task. The experimenter verified the accuracy of the recalled sequence. If correct, the experimenter pressed a key to proceed. If incorrect, the sequence (along with the corresponding classification and verification tasks) was repeated at the end of the experiment. After the experimenter’s key press, “Richtig!” (“Correct!”; in green) or “Falsch!” (“Wrong!”; in red) appeared in the center of the screen.

After the practice block, participants thus performed 36 experimental blocks (each with their unique sequence) to cover all the four-letter sequences that were generated (see above). In order to obtain equal amounts of data for each participant, blocks in which the sequence was incorrectly recalled in phase 3, were repeated at the end of the 36 blocks. Participants took a break of 3 min after they had completed half of the experiment. Excluding the repetitions of incorrect sequences and practice trials, each participant completed a total of 576 experimental trials (36 blocks × 16 trials per block), with only half of these trials being go-trials (*n* = 288). The entire experimental session lasted approximately 1.5 h.

### Data analyses

#### Preprocessing

Blocks were excluded from the analysis if participants failed to correctly recall the memorized sequence (in this case, the block was repeated to maintain sufficient trial numbers), or if they incorrectly responded in > 50% of the nogo-trials (in this case, the block was not repeated). Such exclusions are based on the assumption that these blocks represent periods of inattentiveness or a temporarily loss of the memorized sequence (Ginsburg et al., 2014; Hartmann et al., 2021). For each participant, the error rate (ER) in go-trials was calculated, where an error was defined as either an absence of response or an incorrect response. Participants whose ER fell more than 2.5 SDs above the sample mean were excluded from further analysis.

Next to ER, the analysis of the SPoARC effects focused on the saccade latencies (i.e. reaction times or RTs) from correct responses in go-trials. Saccade latency or RT was defined as the time between the onset of the central letter and the initiation of the saccade that terminated in the designated lateral response trigger box. Incorrect responses, and trials in which participants did not initiate the response-triggering saccade from the center, were removed. This includes cases in which participants were not looking at the center when the target letter appeared and cases in which they made more than one saccade before entering the correct response box. Log-transformed RTs that were more than 2.5 SDs above each participant’s mean for a given response direction were classified as outliers and removed. Mean log-RTs and ERs were then computed for each participant, response direction and serial position and submitted to regression analysis. Actual RTs were later back-transformed for descriptive report of means and for computing differences in latencies (dRTs).

#### RT analyses

In a first step, mean log-latencies were analyzed using a linear mixed-effects model with fixed effects of experimental conditions and random intercepts for subjects to account for repeated measures. The model included two categorical predictors — orientation (horizontal vs. vertical) and response direction (leftward/upward vs. rightward/downward) — as well as the continuous predictor serial position (ranging from 1 to 4). All main effects and higher-order interactions among these three predictors were included in the model. Crucially, the interaction between serial position and response direction reflects SPoARC effects across spatial axes, and the three-way-interaction indicates whether the SPoARC effect is moderated by orientation (horizontal, vertical). Regarding random effect structure, we aimed to specify a maximal random-effects structure including random intercepts and slopes for all within-subject predictors (Barr et al., 2013). However, due to convergence issues, the structure was pragmatically reduced until the model could be estimated without boundary or singular fit errors. The resulting random-effects structure may therefore differ slightly between models. For the RT analysis, the model included a by-participant random intercept and random slopes for response direction and serial position per participant. This accounts for individual variability in overall response latency, and in the effects of response direction and serial position across participants. The model was fitted using the lme4-package in R (Bates et al., 2015). *F*-statistics (using Satterthwaite’s method of correcting degrees of freedom) are reported.

Consistent with prior research, the SPoARC effects were also evaluated by calculating the regression coefficients (betas) for the dRTs as a function of serial position for each participant. For the horizontal axis, dRT was computed as the difference between rightward and leftward latencies, and for the vertical axis as the difference between downward and upward latencies (Fias et al., 1996; Hartmann et al., 2021). The slopes for the horizontal and vertical SPoARC effects–reflecting the beta values of the dRTs–were statistically tested against zero using one-sample *t*-tests. To evaluate the SPoARC effects, we reported Cohen’s d as a measure of effect size, and Bayes Factors (BFs) as a measure of the strength of evidence, computed using the BayesFactor package in R (Morey et al., 2018). Specifically, we reported the Bayes Factor_10_, which quantifies the relative plausibility of the alternative hypothesis (H_1_) compared to the null hypothesis (H_0_). A BF_10_ greater than 1 suggests evidence in favor of H_1_ (1.1–3.1 = weak/anecdotal; 3–10 = moderate; > 10 = strong), whereas a BF_10_ less than 1 indicates evidence favoring H_0_ (0.9 − 0.33 = weak/anecdotal; 0.33 − 0.01 = moderate; < 0.01 = strong) (Jeffreys, 1939; Kelter, 2020). In a next step, we assessed the correlation between the horizontal and vertical SPoARC slopes to explore individual tendencies to associate serial with spatial positions across different orientations. Unless stated otherwise, pearson correlations are computed. 

#### Error analyses

For analyses on accuracy, go-trials in which participants responded with an incorrect response direction were classified as 1, and go-trials with a correct response as 0. Errors were analyzed using a logistic regression model with the generalized linear mixed effects model (glmer) function from the lme4 package in R (Bates et al., 2015). As for the analysis of RTs, the model included the categorical predictors orientation (horizontal versus vertical) and response direction (left/up versus right/down), the continuous predictor serial position (ranging from 1 to 4), and all higher-order interactions. The random effect structure included a random intercept and random slopes for response direction, serial position and orientation by participant. Again, the interaction between response direction and serial position reflect SPoARC effects across orientations, and the three-way interaction indicates whether the SPoARC effect is moderated by orientation (horizontal vs. vertical). Estimates and standard error of the estimates (Std. error) were reported, and Wald *z*-test was used to compute *p*-values for the estimates. Sum coding was used for the contrasts of the categorical variables, and the continuous variable serial position was mean-centered.

In analogy to the RT analysis, we also evaluated the SPoARC effect by calculating the regression coefficients (betas) for the dERs as a function of serial position for each participant using one-sample *t*-tests, and computed the correlation between horizontal and vertical SPoARC effects.

#### Inverse efficiency analyses

Because response times and error rates capture partially distinct aspects of task performance and may show dissociable effects, we additionally report the inverse efficiency score (IES) as a combined performance measure. The IES accounts for speed–accuracy trade-offs (see Appendix A for confirmation of these) by adjusting response times for accuracy (Bruyer & Brysbaert, 2011). IES were computed by dividing mean RTs for correct responses by the proportion of correct responses. Lower IES reflect better performance than higher IES. IES were analyzed using the same linear mixed-effects models as described above for RTs. The model included a by-participant random intercept; adding random slopes led to boundary (singular) fits. Analyses were complemented by dIES and by correlating participants’ individual regression slopes (beta coefficients) for the horizontal and vertical orientation.

### Transparency and openness

We report how we determined our sample size, all data exclusions, all manipulations, and all measures in the study. All data, analysis code, and research materials are available at https://osf.io/6krq9/. We have complied with APA ethical standards in the treatment of participants and their data. This study’s design and its analysis were not pre-registered.

## Results

A total of five blocks (0.4% of all blocks) were repeated across all participants at the end of experiment due to participants’ incorrect recollection of the memory sequence. Three blocks (0.2% of all blocks) were removed in total across all participants because participants responded in 50% or more of the no-go trials. One participant with a high ER for go-trials (32.6%; more than 2.5 SDs above the sample mean) was excluded from further analysis, leaving the data of *n* = 33 participants to enter the analyses below. A total of 879 error go-trials (9.2% of go-trials) were removed for the analysis of saccade latencies (112 due to no response, and 767 due to incorrect response), as well as 520 trials in which participants did not start the response triggering saccade from the center (5.5% of go-trials). Finally, 134 trials with log-latencies exceeding 2.5 SDs of each participant’s mean per response direction were excluded from analysis (1.4% of go-trials).

### RTs

**Fig. 2:**
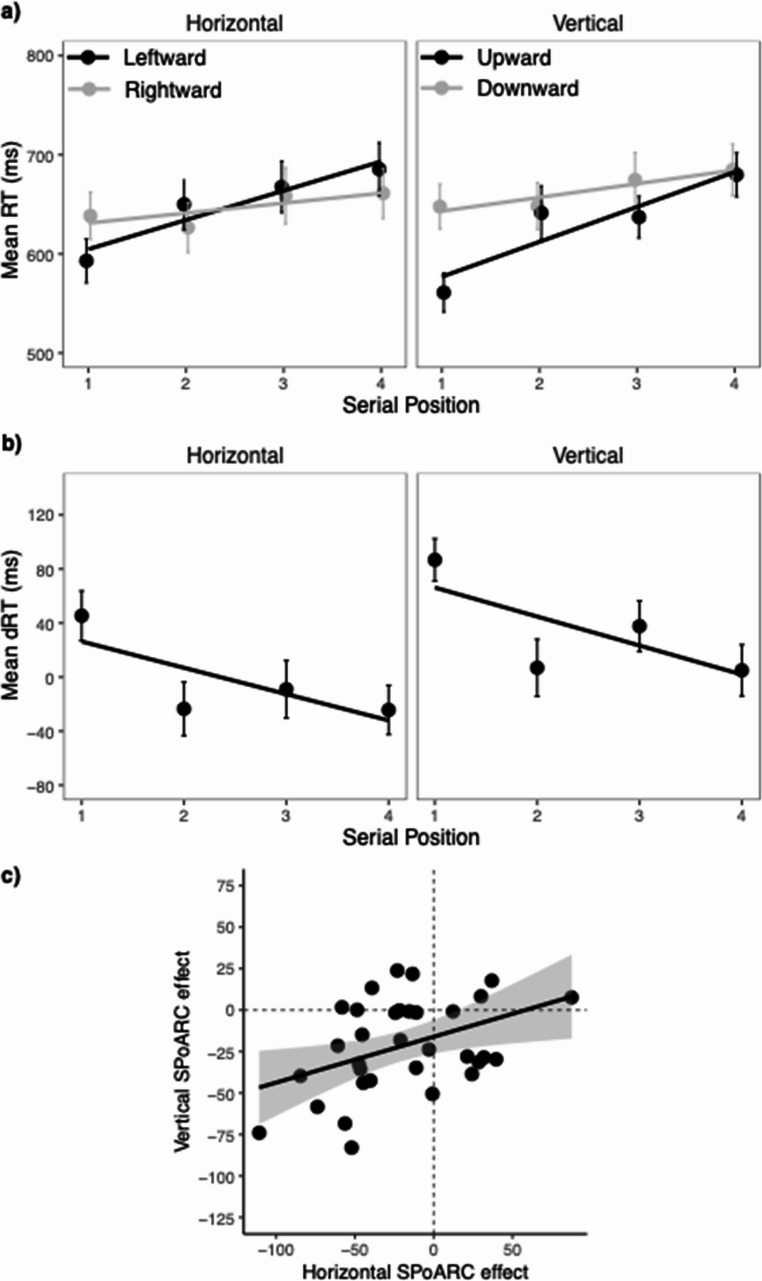
Mean RTs, mean dRTs and correlation between horizontal and vertical SPoARC effect in Experiment 1. The upper panel (**a**) shows mean response times (RTs) for each response direction and serial position. The middle panel (**b**) shows mean dRT (horizontal: right minus left; vertical: down minus up) for each serial position. Error bars depict +/- 1 SEM. The lower panel (**c**) shows the correlation between the horizontal and vertical SPoARC effect (individual regression slopes of dRTs as a function of serial position). The solid line shows the linear fit (with +/- 95 CI). The dashed lines mark the boundaries for positive and negative SPoARC effects, with dots in the lower left quadrant reflecting the concurrent presence of early-leftward/ and early-upward associations

Mean RTs, back-transformed from log-latencies, for each serial position and response direction are presented in the upper panel of Fig. 2a, and the results of the analysis of log-latencies are presented in Table 2. The analysis revealed a significant effect of serial position, showing faster responses at earlier serial positions (*M*_1_ = 610, *SEM* = 19, *M*_2_ = 642, *SEM* = 23, *M*_3_ = 660, *SEM* = 23, *M*_4_ = 678, *SEM* = 22). Most importantly, there was a significant interaction between serial position and response direction, indicating a SPoARC effect across orientations. As illustrated in Fig. 2a, participants responded faster with leftward saccades at early serial positions but faster with rightward saccades at later serial positions. For the vertical spatial axis, participants generally responded faster with upward saccades compared to downward saccades, but this difference reduced across serial positions. Τhe SPoARC effect did not interact with orientation (*F* < 1), indicating that the strength of the SPoaARC effect was similar for the horizontal and vertical orientation.

**Table 2:** Summary of RT analyses for Experiment 1

Response direction (RD)	F	*P*
	2.41	0.130
Serial position (SP)	43.36	< 0.001
Orientation (O)	< 0.00	0.983
RD x SP (SPoARC effect)	21.45	< 0.001
RD x O	12.18	< 0.001
SP x O	1.25	0.263
RD x SP x O	0.27	0.605
Horizontal orientation		
RD	0.05	0.828
SP	46.11	< 0.001
RL x SP (SPoARC effect)	13.68	< 0.001
Vertical orientation		
RD	5.76	0.022
SP	28.27	< 0.001
RL x SP (SPoARC effect)	16.66	< 0.001

There was also a significant interaction between response direction (leftward/upward, rightward/downward) and orientation (horizontal, vertical). To further disentangle this interaction, and also to further confirm the presence of the SPoARC effect for both orientations, the analysis was repeated separately for the horizontal and vertical orientations (see Table 1). The interaction between response direction and serial position (SPoARC effect) was significant in both orientations (*p*s < 0.001). The separate analyses also revealed a main effect of response direction for the vertical orientation, with faster responses for upward (*M* = 630, *SEM* = 20) compared to downward responses (*M* = 664, *SEM* = 23). This replicates the well documented upward bias of faster upward (vs. downward) saccades (cf. Abegg et al., 2015; Greene et al., 2014, 2020). In contrast, and similar to previous work, no difference between leftward (*M* = 649, *SEM* = 24) and rightward (*M* = 646, *SEM* = 24) responses was present in the horizontal orientation.

Figure 2b displays the mean dRTs. Analysis of the individual regression coefficients (betas) for the horizontal dRTs as a function of serial position confirmed a significant SPoARC effect, *t*(32) = −2.66, *p* =.012, Cohen’s *d* = −0.46. The mean slope was − 19 ms (*SEM* = 7). The associated BF_10_ of 3.70 suggests moderate evidence supporting the presence of an SPoARC effect. Similarly, there was a significant vertical SPoARC effect, *t*(32) = −4.42, *p* <.001, Cohen’s *d* = −0.77. The mean slope was − 21 ms (*SEM* = 5). The BF_10_ of 241.91 indicates strong support for the vertical SPoARC effect. Importantly, there was a significant positive correlation between the horizontal and vertical SPoARC slopes (*r* =.42, *p* =.016, BF_10_ = 4.69), suggesting that participants who exhibit a stronger early-left and late-right association are also more likely to display an early-up and late-down association (see Fig. 2c).

### Error rates

**Fig. 3:**
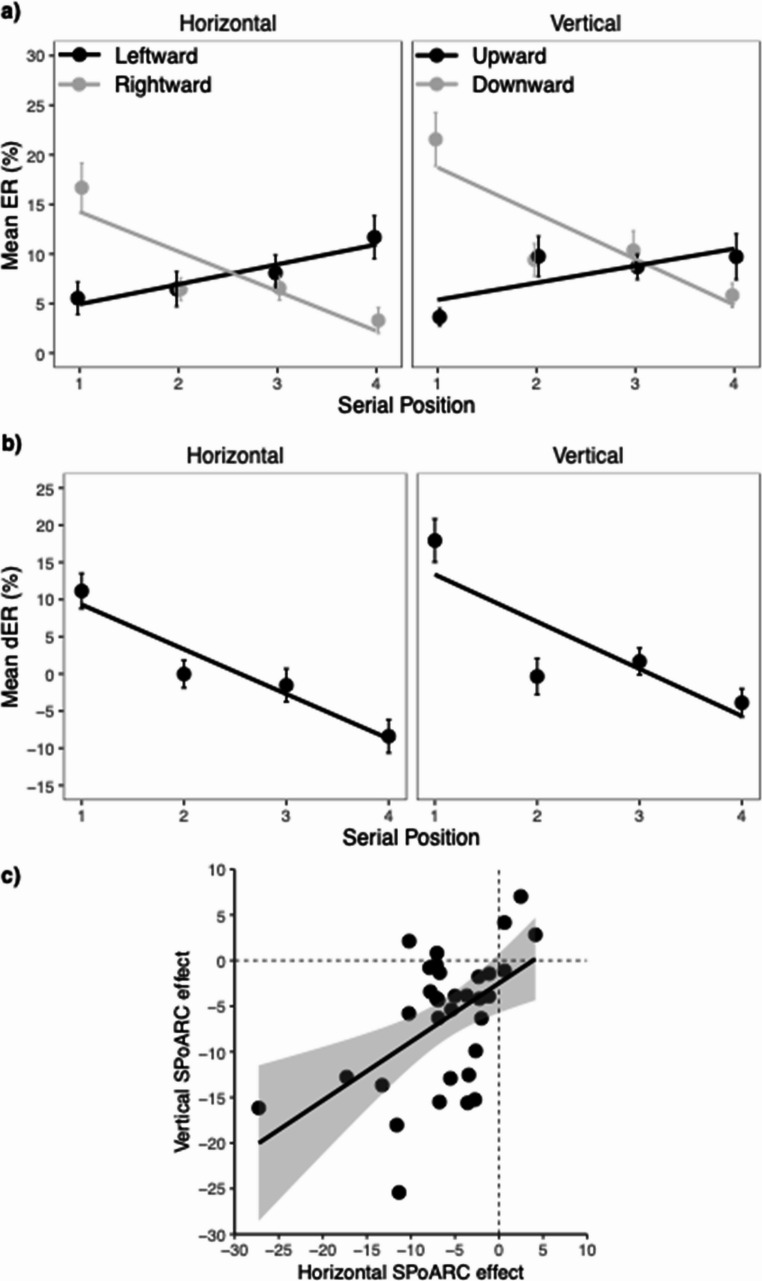
Mean ERs, mean dERs and correlation between horizontal and vertical SPoARC effect in Experiment 1. The upper panel (**a**) shows mean error rates (ERs) for each response direction and serial position. The middle panel (**b**) shows mean dER (horizontal: rightward minus leftward; vertical: downward minus upward) for each serial position. Error bars depict +/- 1 SEM. The lower panel (**c**) shows the correlation between the horizontal and vertical SPoARC effect (individual regression slopes of dERs as a function of serial position). The solid line shows the linear fit (with +/- 95 CI). The dashed lines mark the boundaries for positive and negative SPoARC effects, with dots in the lower left quadrant reflecting the concurrent presence of early-leftward/upward and laterightward/downward associations

Mean ER for each serial position and response direction is depicted in Fig. 3, and the results of the glmer logistic regression analysis are summarized in Table 3. The analysis revealed a significant linear effect of serial position, with decreasing ERs especially at the first serial position as compared to further serial positions (*M*_1_ = 11.7%, *SEM* = 1.3, *M*_2_ = 7.9%, *SEM* = 1.1, *M*_3_ = 8.3%, *SEM* = 1.0, *M*_4_ = 7.6%, *SEM* = 1.3). Moreover, ERs were significantly higher for vertical (*M* = 9.7%, *SEM* = 1.1) compared to horizontal responses (*M* = 8.1%, *SEM* = 1.2), and also significantly higher for trials that required a rightward or downward response (*M* = 9.9%, *SEM* = 1.1) compared to a leftward and upward response (*M* = 7.9%, *SEM* = 1.2). response direction and orientation interacted, suggesting that the difference between leftward/upward and rightward/downward responses were driven by vertical responses (see separate analysis for horizontal and vertical orientation).

**Table 3:** Summary of ER analyses for Experiment 1

	Estimate (Std. Error)	z-value	*P*
Combined spatial orientations			
Response direction (RD)	−0.13 (0.06)	−1.96	0.050
Serial position (SP)	−0.16 (0.04)	−3.57	< 0.001
Orientation (O)	−0.16 (0.06)	−2.59	0.009
RD x SP (SPoARC effect)	0.42 (0.04)	10.99	< 0.001
RD x O	0.12 (0.04)	2.78	0.005
SP x O	−0.02 (0.04)	−0.62	0.538
RD x SP x O	0.04 (0.04)	1.01	0.315
Horizontal orientation			
RD	−0.06 (0.10)	−0.63	0.527
SP	−0.15 (0.05)	−2.78	0.005
RD x SP (SPoARC effect)	0.46 (0.05)	8.40	< 0.001
Vertical orientation			
RD	−0.24 (0.09)	−2.81	0.005
SP	−0.11 (0.05)	−2.25	0.024
RD x SP (SPoARC effect)	0.38 (0.05)	7.63	< 0.001

Results are based on generalized linear mixed effects model at the trial level, with 1 = error and 0 = correct. Estimates reflect log odds

Most importantly, there was a significant interaction between serial position and response direction, indicating a SPoARC effect across both orientations also for ERs. Specifically, participants made more errors in SPoARC incongruent trials (leftward and upward responses to late serial positions and rightward and downward responses to early serial positions) compared to SPoARC congruent trials (leftward and upward responses to early serial positions and rightward and downward responses to late serial positions; see Fig. 3a). Τhis SPoARC effect did not interact with orientation, indicating that the effect was comparable across both orientations. Nevertheless, to confirm the presence of the SPoARC effect within each orientation, separate analyses were conducted for the horizontal and vertical orientations. The separate analyses of the horizontal and vertical orientations confirmed the presence of both SPoARC effects (see Table 3). For the vertical orientation, there was also significant effect of response direction, with higher ERs for downward (*M* = 11.7%, *SEM* = 1.4) compared to upward responses (*M* = 7.9%, *SEM* = 1.3), while no difference was found between leftward (*M* = 7.9%, *SEM* = 1.5) and rightward (*M* = 8.2%, *SEM* = 1.2) responses. Thus, participants made more incorrect upward responses in trials that required a downward response, in line with the previously mentioned upward bias.

Differences in ERs (dERs) are shown in Fig. 3b. Analysis of the individual regression coefficients (betas) for the horizontal dERs as a function of serial position confirmed a significant SPoARC effect, *t*(32) = −5.80, *p* <.001, Cohen’s *d* = −1.01. The mean slope was − 6.0% (*SEM* = 1.0). The associated BF_10_ of 9370.06 suggests strong evidence supporting the presence of an SPoARC effect. Similarly, there was a significant vertical SPoARC effect, *t*(32) = −4.95, *p* <.001, Cohen’s *d* = −0.86. The mean slope was − 6.3% (*SEM* = 1.3). The BF_10_ of 967.69 indicates strong support for the vertical SPoARC effect. Finally, there was a significant positive correlation between the horizontal and vertical SPoARC slopes (*r* =.52, *p* =.002, BF_10_ = 23.79), suggesting that participants who exhibit a stronger early-leftward and late-rightward association are also more likely to display an early-upward and late-downward association (see Fig. 3c).

### IES

**Fig. B1:**
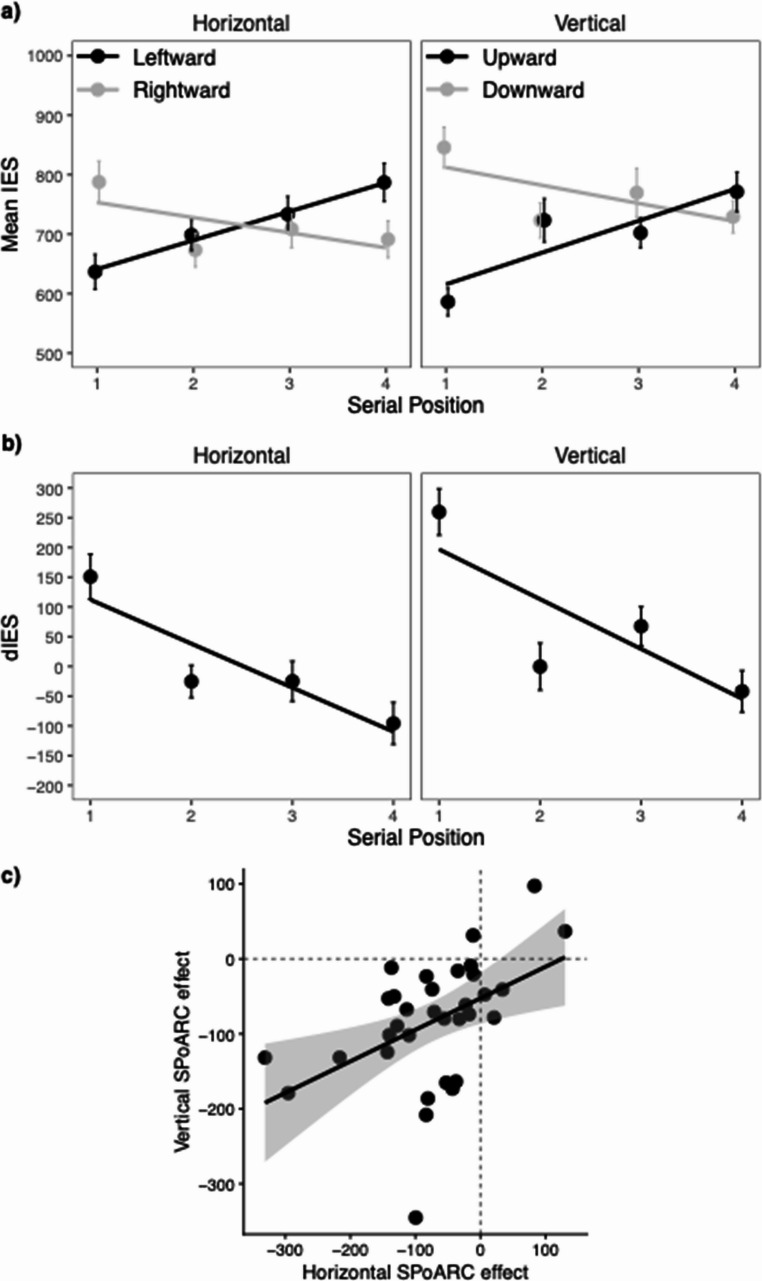
Mean IES, mean dIES and correlation between horizontal and vertical SPoARC effect in Experiment 1. The upper panel (**a**) shows mean IES for each response direction and serial position. The middle panel (**b**) shows mean dIES (horizontal: right minus left; vertical: down minus up) for each serial position. Error bars depict +/- 1 SEM. The lower panel (**c**) shows the correlation between the horizontal and vertical SPoARC effect (individual regression slopes of dIES as a function of serial position). The solid line shows the linear fit (with +/- 95 CI). The dashed lines mark the boundaries for positive and negative SPoARC effects, with dots in the lower left quadrant reflecting the concurrent presence of early-leftward/and early-upward associations

The analysis of IES revealed a significant effect of response direction, *F*(1, 488) = 10.34, *p* =.001, with better performance (i.e., lower IES) for leftward/upward responses (*M* = 705, *SEM* = 23) compared to rightward/downward responses (*M* = 741, *SEM* = 26) and also a significant effect of serial position, *F*(1, 488) = 5.27, *p* =.022, showing lower performance with increasing serial positions (*M*_*1*_ = 714, *SEM* = 22, *M*_*2*_ = 705, *SEM* = 24, *M*_*3*_ = 728, *SEM* = 27, *M*_*4*_ = 745, *SEM* = 23). Most importantly, there was a significant interaction between serial position and response direction, *F*(1, 488) = 61.17, *p* < 001, indicating the SPoARC effect across orientations. Τhe SPoARC effect did not interact with orientation (*F* < 1). Further analysis of the individual regression coefficients (betas) for the dIES as a function of serial position confirmed both a significant horizontal SPoARC effect, *t*(32) = −4.54, *p* <.001, Cohen’s *d* = −0.79 (mean slope of −74ms; *SEM* = 16), and a significant vertical SPoARC effect, *t*(32) = −5.76, *p* <.001, Cohen’s *d* = −1.00 (mean slope of −84ms; *SEM* = 15). The associated BF_10_ of 329 and 8415, respectively, indicated strong support for the presence both SPoARC effects. Finally, there was also a significant correlation between the horizontal and vertical SPoARC slopes (*r* =.41, *p* =.006, BF_10_ = 10.33). Mean IES, dIES and the correlation are illustrated in Figure B1, and full statistical report can be found in Table B1 in Appendix B.

## Discussion

The current study is the first to demonstrate the joint occurrence of two differently oriented SPoARC effects in the same task. Whereas Hartmann et al. (2021) demonstrated horizontal and vertical SPoARC effects to occur within the same sample but across different sessions, here the horizontal and vertical SPoARC effects were obtained within-blocks, with orientation varying from trial to trial. In line with the previous study by Hartmann et al. (2021), here, too, a positive correlation was observed between horizontal and vertical SpoARC effects. These findings fit well the multidimensional scenario outlined in the Introduction (see also Table 1), indicating that spatial coding on the mental whiteboard is flexible enough to cope with the trial-by-trial uncertainty in the orientation of response mapping. Moving beyond the use of unidimensional mental lines, the findings indicate that the task context probed participants either to encode the sequence along a single, diagonally oriented mental line that can be used for both horizontal and vertical trials, or to encode and maintain both a horizontally and a vertically oriented order representation. In each of these strategies, both the horizontal and vertical axes are exploited simultaneously to support serial order ability – providing the first support for multidimensional coding on the mental whiteboard (cf. Aleotti et al., 2023 for similar conclusions on spatial-numerical associations).

Additionally, the top-to-bottom oriented vertical SPoARC effect is an important replication with respect to the functional role of spatial coding in serial order: Each of the possible epiphenomenal accounts (polarity coding, metaphor theory, and indirect spatial-numerical association logic) that we know from the literature, would predict a bottom-to-top mapping. As such, next to the demonstration of multidimensional coding (indicating goal-directed coding on the mental whiteboard), replicating here the observation by Hartmann et al. (2021) that vertical coding occurs in a top-to-bottom mapping adds support against the notion that spatial coding for serial order in verbal working memory is merely an epiphenomenon.

### Experiment 2

If the multidimensional coding observed in Experiment 1 is a *proactive* response from a goal-directed mechanism that builds context-appropriate representations for efficient serial order performance (i.e. cognitive control; e.g. Braver, 2012), then we would expect that it can be abolished (or at least reduced) by dedicated changes to the task context. Experiment 2 was set up as a conceptual replication of Experiment 1, with one major alteration in the response mappings. While the response orientation (horizontal, vertical) in Experiment 1 varied from trial to trial, the response mapping was fixed for each participant in Experiment 1: A vowel (consonant) always required a left (right) or up (down) response for the same participant, emphasizing a close connection between left and up, and right and down (i.e. diagonal). Experiment 2 introduces response uncertainty even further by also varying the response mapping from trial to trial. Rather than having the vowel-response and consonant-response boxes being systematically mapped on the left/up and right/down locations per participant, the vowel- and consonant-responses were now mapped onto locations randomly across trials. Specifically, the precise mapping was cued within each trial just before the target letter and response boxes appeared on the screen. Hence, whereas phases 1 and 3 of the blocks were the same as in Experiment 1, the classification task in phase 2 was now containing an extra cuing step: Just before the target letter and the response boxes appeared, a color cue (see Method section below for details) provided information about both the upcoming trial’s orientation (horizontal versus vertical), and the precise location of the vowel and consonant response boxes.^5^

We anticipated this methodological adjustment to negatively affect multidimensional coding for two (related) reasons. First, it is possible that the fixed response mapping in Experiment 1, in which top/left and bottom/right always coincided by being mapped onto the same response category (i.e. vowel or consonant), served as a contextual trigger for participants to exploit an encoding strategy in which a single, diagonally oriented mental line (i.e. from top-left to right-bottom) was proactively set for serial order processing. If this reasoning is correct, then introducing response mapping uncertainty (i.e., varying the mapping from trial to trial) that breaks the systematic associations between top-left and bottom-right, is predicted to reduce the efficiency of diagonal coding – and thus potentially break down the tendency to exploit diagonal lines. Second, the new design added cognitive demands to the task. Given that controlled processes require more mental effort (e.g. Shenhav et al., 2017), such enhanced cognitive load is expected to reduce the cognitive resources left for mental operations related to the simultaneous exploitation of and switching between multiple order representations. Overall, then, whether the multidimensional coding observed in Experiment 1 was due to diagonal or multiple order representations, the adjusted design with varying response mappings is predicted to reduce (the tendency for) multidimensional coding – effectively pushing participants back to one of the other scenarios from Table 1 (i.e. the unidimensional scenarios or the no-spatialization scenario).

### Methods

#### Participants

To define the minimum sample size for Experiment 2, we used the smallest effect size observed in Experiment 1 as a reference (*d* = 0.46). This yielded a required sample size of 40 participants to detect an effect of *d* ≥ 0.46 with a power of > 0.80, assuming a two-tailed test and a maximum Type I error rate of α = 0.05 (G*Power 3; Faul et al., 2007). As such, 44 participants were recruited. All were undergraduate students who participated in exchange for course credit (mean age = 21.2, ranging from 19 to 34; 36 female). None of them participated in Experiment 1. All participants reported German as their native language.

#### Stimuli and procedure

**Fig. 4:**
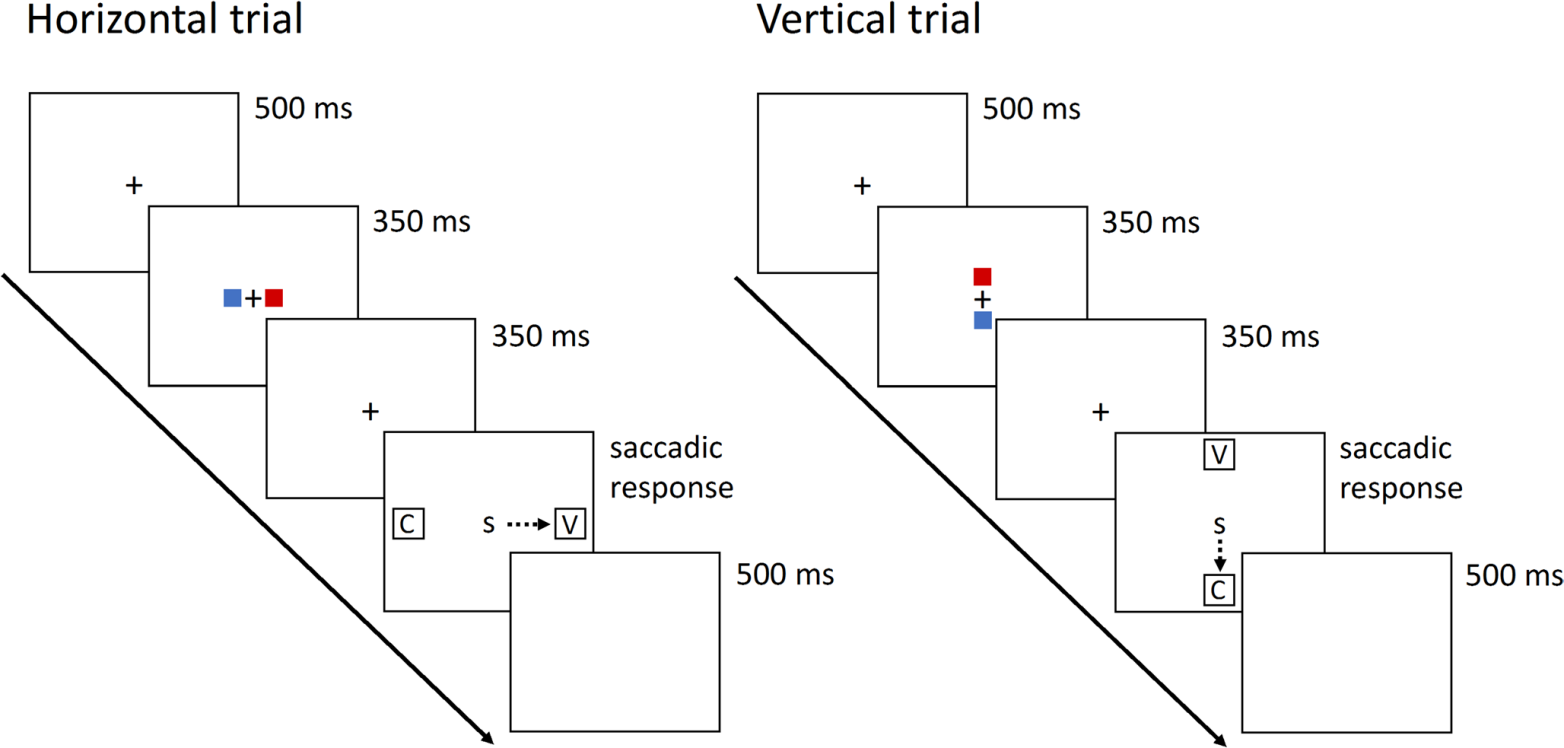
Trial example of the consonant-vowel-classification task in Experiment 2 for a participant for whom the red color indicated the vowel response box location, and the blue color indicated the consonant response box location

The stimuli and procedure were identical to Experiment 1, with the following modifications in the consonant-vowel classification task (phase 2). The positions of the consonant and vowel response boxes were cued before the target (and saccade response boxes) came onto the screen, by red and blue squares (10 × 10 pixels) that were positioned 40 pixels to the left and right (horizontal trials) or above and below (vertical trials) the central fixation cross for 350 ms. The color cues were implemented to prevent participants from making eye movements toward the response boxes to read the labels (C or V), ensuring that eye movements were solely for response. For half of the participants, the blue cue indicated the position of the consonant response box, while the red cue indicated the position of the vowel response box; the reversed color coding was applied to the other half of participants. Following an additional 350 ms, during which only the central fixation cross was visible, the target and response boxes appeared and remained on the screen until participants responded or 3000 ms elapsed. Examples of the trial procedure of Experiment 2 are depicted in Fig. 4. Within each consonant-vowel-classification task (8 go-trials), each response direction (right, left, up, down) was required once per letter category (consonant or vowel), and an equal number of responses were collected for each location per serial position across the entire experiment.

#### Data analyses

Data analysis was identical to Experiment 1, with mixed-effects models including the fixed effect structure of response direction (leftward/upward, rightward/downward), serial position (1–4), and orientation (horizontal, vertical), along with their interactions. For the RT analysis, the maximum random effect structure included a random intercept and random slopes for response direction and serial position by participant. For the ER analysis, the maximum random effect structure included a random intercept and random slopes for response direction, serial position and orientation by participant. Again, dRT and dER were analyzed as well as the correlations between the horizontal and vertical SPoARC effects. Like in Experiment 1, IES were also analyzed. Since a small number of extreme IES values were observed here, dIES analyses were complemented by Wilcoxon signed-rank tests, and slope correlations were complemented by a Spearman rank correlation test.

## Results

Data from one participant were excluded from the analysis due to a high error rate (45.8% errors in go-trials; more than 2.5 SDs above the sample mean), leaving the data of *n* = 43 participants to enter the analyses below. Twenty blocks (1.3% of all blocks) were repeated at the end of the experiment due to participants’ incorrect recollection of the memory sequence. Additionally, 7 blocks (0.5% of all blocks) were removed from analyses because participants responded in 50% or more of the nogo-trials. A total of 1,906 error go-trials (15.5% of go-trials) were removed for the analysis of saccade latencies (218 due to no response, and 1688 due to incorrect response), as well as 818 trials in which participants did not start the response triggering saccade from the center (6.6% of go-trials). Finally, 162 trials with log-latencies exceeding 2.5 SDs of each participant’s mean per response direction were excluded from analysis (1.3% of go-trials).

### RTs

As expected, the variable stimulus-response mapping led to a significant increase in RTs in Experiment 2 (*M* = 859, *SEM* = 36) compared to Experiment 1 (*M* = 647, *SEM* = 21), as confirmed by an independent sample *t*-test, *t*(74) = −4.82, *p* <.001, Cohen’s *d* = −1.14.

**Fig. 5:**
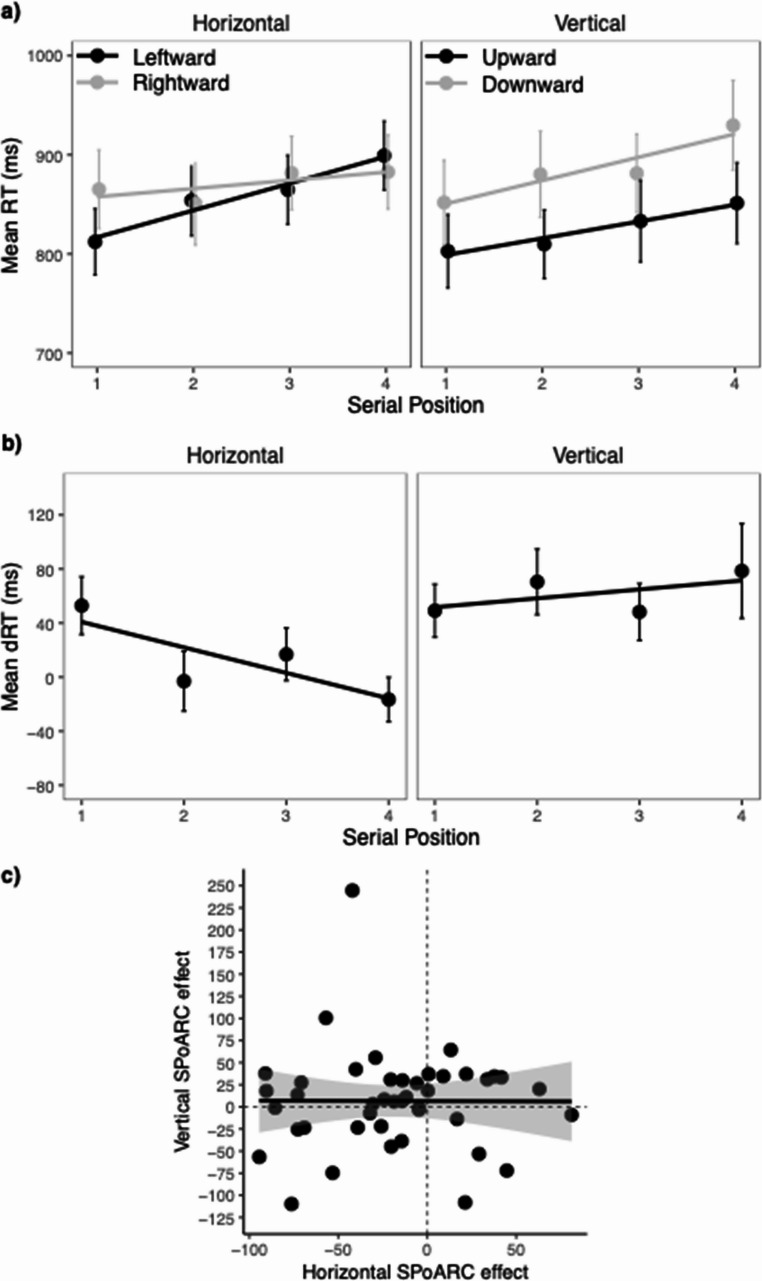
Mean RTs, mean dRTs and correlation between horizontal and vertical SPoARC effect in Experiment 2. The upper panel (**a**) shows mean response times (RTs) for each response direction and serial position. The middle panel (**b**) shows mean dRT (horizontal: right minus left; vertical: down minus up) for each serial position. Error bars depict +/- 1 SEM. The lower panel (**c**) shows the correlation between the horizontal and vertical SPoARC effect (individual regression slopes of dRTs as a function of serial position). The solid line shows the linear fit (with +/- 95 CI). The dashed lines mark the boundaries for positive and negative SPoARC effects, with dots in the lower left quadrant reflecting the concurrent presence of early-leftward/late-rightward and earlyupward/late-downward associations

Mean RTs, back-transformed from log-latencies, for each serial position and response direction for Experiment 2 are presented in Fig. 5a, and the results of the analysis of log-latencies is reported in 5.

The analysis of log-latencies of Experiment 2 revealed a significant effect of serial position, showing faster responses at earlier serial positions (*M*_1_ = 833, *SEM* = 36, *M*_2_ = 849, *SEM* = 37, *M*_3_ = 865, *SEM* = 36, *M*_4_ = 891, *SEM* = 36). There was also a significant effect of response direction, with faster leftward/upward responses (*M* = 841, *SEM* = 34) compared to rightward/downward responses (*M* = 878, *SEM* = 39), and also an effect of orientation, with faster vertical (*M* = 855, *SEM* = 38) compared to horizontal responses (*M* = 864, *SEM* = 35). Orientation interacted with response direction, indicating that the advantage of the leftward/upward (vs. rightward/downward) responses is driven by the vertical orientation, as can be derived from Fig 5a, and from the separate analysis for the orientations (Table 4). Most importantly, there was no significant interaction between serial position and response direction (*F* < 1), indicating no SPoARC effect across orientations. However, the SPoARC effect significantly interacted with orientation, which is further addressed by separate analysis of horizontal and vertical orientation.

**Table 4:** Summary of RT analyses for Experiment 2

	F	*P*
Combined spatial orientations		
Response direction (RD)	5.39	0.025
Serial position (SP)	38.66	< 0.001
Orientation (O)	6.90	0.009
RD x SP (SPoARC effect)	0.75	0.387
RD x O	17.95	< 0.001
SP x O	< 0.01	0.939
RD x SP x O	5.42	0.020
Horizontal orientation		
RD	0.24	0.628
SP	34.98	< 0.001
RD x SP (SPoARC effect)	7.37	0.007
Vertical orientation		
RD	9.76	0.003
SP	26.24	< 0.001
RD x SP (SPoARC effect)	1.11	0.294

The separate analysis for the horizontal and vertical orientations revealed a significant SPoARC effect for the horizontal but not for the vertical orientation (see Table 5). Specifically, for the horizontal orientation, participants responded faster with leftward saccades at the early serial position and faster with rightward saccades at later serial positions (see Fig. 5a). For the vertical spatial axis, participants generally responded faster with upward saccades (*M* = 824, *SEM* = 37) compared to downward saccades (*M* = 886, *SEM* = 42) but there was no interaction with serial position (SPoARC effect).

dRTs are shown in Fig. 5b. Analysis of the individual regression coefficients (betas) for the dRTs as a function of serial position revealed a significant horizontal SPoARC effect, *t*(42) = −2.84, *p* =.007, Cohen’s *d* = −0.43. The associated BF_10_ of 5.41 suggests moderate evidence supporting the horizontal SPoARC effect. The mean slope was − 19 ms (*SEM* = 7). In contrast, the vertical SPoARC slope was not significant, *t*(42) = 0.76, *p* =.454, with an effect size of Cohen’s *d* = 0.12. The mean slope was 7 ms (*SEM* = 9). The BF_10_ of 0.22 indicates moderate support for the absence of a vertical SPoARC effect. Finally, there was no correlation between the horizontal and vertical SPoARC slopes (*r* <.01, *p* =.981, BF_10_ = 0.34; see Fig. 5c).

### Error rates

**Fig. 6:**
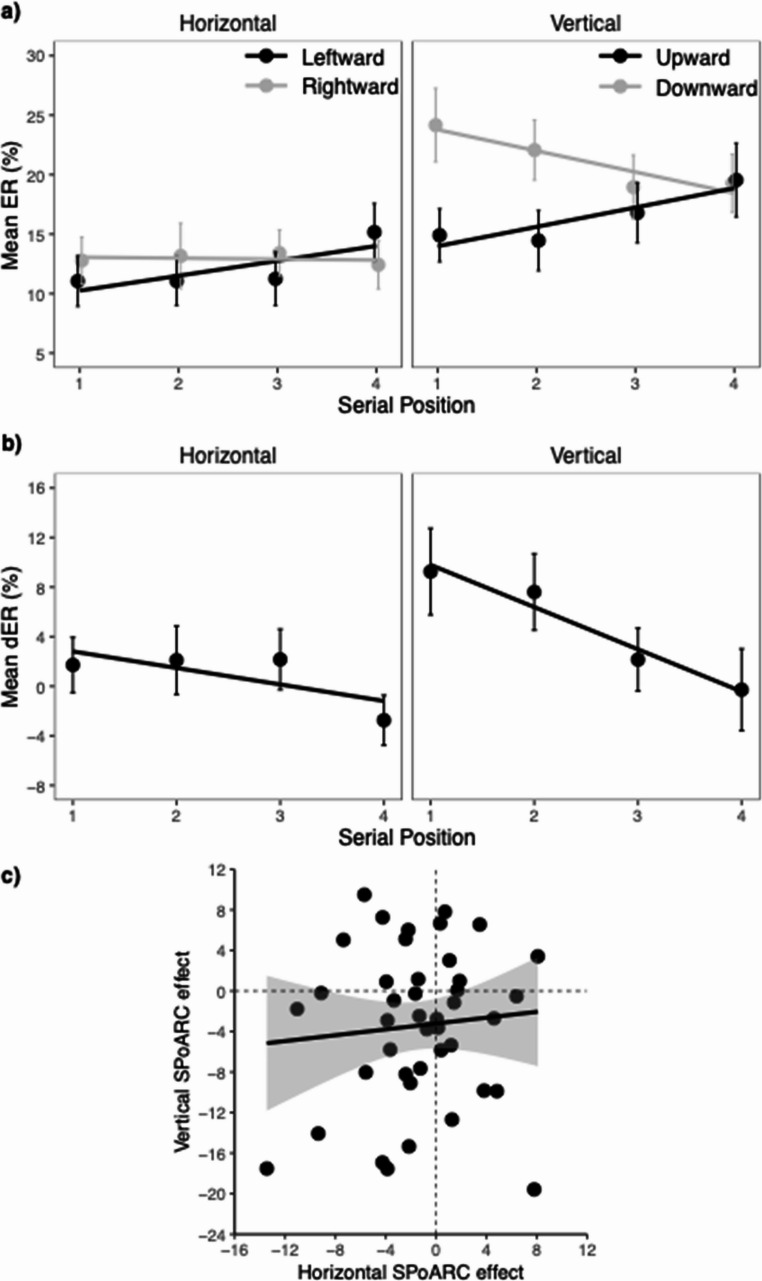
Mean ERs, mean dERs and correlation between horizontal and vertical SPoARC effect in Experiment 2. The upper panel (**a**) shows mean error rates (ERs) for each response direction and serial position. The middle panel (**b**) shows mean dER (horizontal: rightward minus leftward; vertical: downward minus upward) for each serial position. Error bars depict +/- 1 SEM. The lower panel (**c**) shows the correlation between the horizontal and vertical SPoARC effect (individual regression slopes of dERs as a function of serial position). The solid line shows the linear fit (with +/- 95 CI). The dashed lines mark the boundaries for positive and negative SPoARC effects, with dots in the lower left quadrant reflecting the concurrent presence of early-leftward/late-rightward and early-upward and late-downward associations

As expected, the variable stimulus-response mapping led to a significant increase in ERs in Experiment 2 (*M* = 15.4%, *SEM* = 1.7) compared to Experiment 1 (*M* = 8.9%, *SEM* = 1.1), as confirmed by an independent sample *t*-test, *t*(74) = −3.00, *p* =.004, Cohen’s *d* = −0.72. Mean error rates for each serial position and response direction for Experiment 2 is depicted in Fig. 6, and the results of the glmer logistic regression analysis are summarized in Table 5.

**Table 5:** Summary of ER analyses for experiment 2

	Estimate (Std. Error)	z-value	*p*
Combined spatial orientations			
Response direction (RD)	−0.15 (0.06)	−2.24	0.025
Serial position (SP)	0.03 (0.03)	0.80	0.425
Orientation (O)	−0.31 (0.05)	−6.00	< 0.001
RD x SP (SPoARC effect)	0.10 (0.03)	3.68	< 0.001
RD x O	0.06 (0.03)	2.19	0.029
SP x O	0.03 (0.03)	1.12	0.264
RD x SP x O	−0.03 (0.03)	−1.12	0.264
Horizontal orientation			
RD	−0.07 (0.08)	−0.88	0.381
SP	0.06 (0.04)	1.57	0.118
RD x SP (SPoARC effect)	0.06 (0.04)	1.67	0.095
Vertical orientation			
RD	−0.23 (0.08)	−2.83	0.005
SP	< 0.01 (0.03)	0.03	0.978
RD x SP (SPoARC effect)	0.12 (0.03)	3.63	< 0.001

Results are based on generalized linear mixed effects model at the trial level, with 1 = error and 0 = correct. Estimates reflect log odds

The analysis revealed a significant effect of orientation, with a higher error rate for vertical (*M* = 18.6%, *SEM* = 1.9) compared to horizontal trials (*M* = 12.4%, *SEM* = 1.6). There was also a significant effect of response direction, with a higher error rate for trials requiring correct rightward/downward (*M* = 16.9%, *SEM* = 2.0) compared to leftward/upward responses (*M* = 13.9, *SEM* = 1.9). Response direction interacted with orientation, indicating that the higher error rate for rightward/downward responses is driven by downward trials of the vertical orientation (see Fig. 6 and separate analysis for vertical orientation). Most importantly, there was a significant interaction between serial position and response direction, indicating a SPoARC effect across orientations for ERs. The SPoARC effect did not interact with Orientation.

The separate analysis for the horizontal and vertical orientations showed that the horizontal SPoARC effect did not reach significance, while a significant vertical SPoARC effect was observed in ERs. Specifically, for the horizontal orientation, participants made more errors in SPoARC incongruent (early-rightward, late-leftward) than SPoARC congruent (early-leftward, late-rightward) trials (see Fig. 6a). For the vertical orientation, participants generally made more errors in trials which required a downward response (*M* = 21.2%, *SEM* = 2.4) compared to upward responses (*M* = 16.3%, *SEM* = 2.2), meaning that they made more incorrect upward responses. Crucially, this upward bias was strongest for earlier serial positions, reflecting that earlier serial positions induced a higher tendency to make an upward response – in line with a SPoARC effect.

dERs are shown in Fig. 6b. Analysis of the individual regression coefficients (betas) for dERs as a function of serial position confirmed a trend for a horizontal SPoARC effect, *t*(42) = −1.88, *p* =.067, Cohen’s *d* = −0.29. The mean slope was − 1.3% (*SEM* = 0.7). The associated BF_10_ of 0.82 suggests inconclusive evidence. There was a significant vertical SPoARC effect, *t*(42) = −2.94, *p* =.005, Cohen’s *d* = −0.45. The mean slope was − 3.4% ms (*SEM* = 1.2). The BF_10_ of 6.87 supports moderate evidence for the vertical SPoARC effect. Finally, there was no significant correlation between the horizontal and vertical SPoARC slopes (*r* =.09, *p* =.573, BF_10_ = 0.39).

### IES

**Fig. B2:**
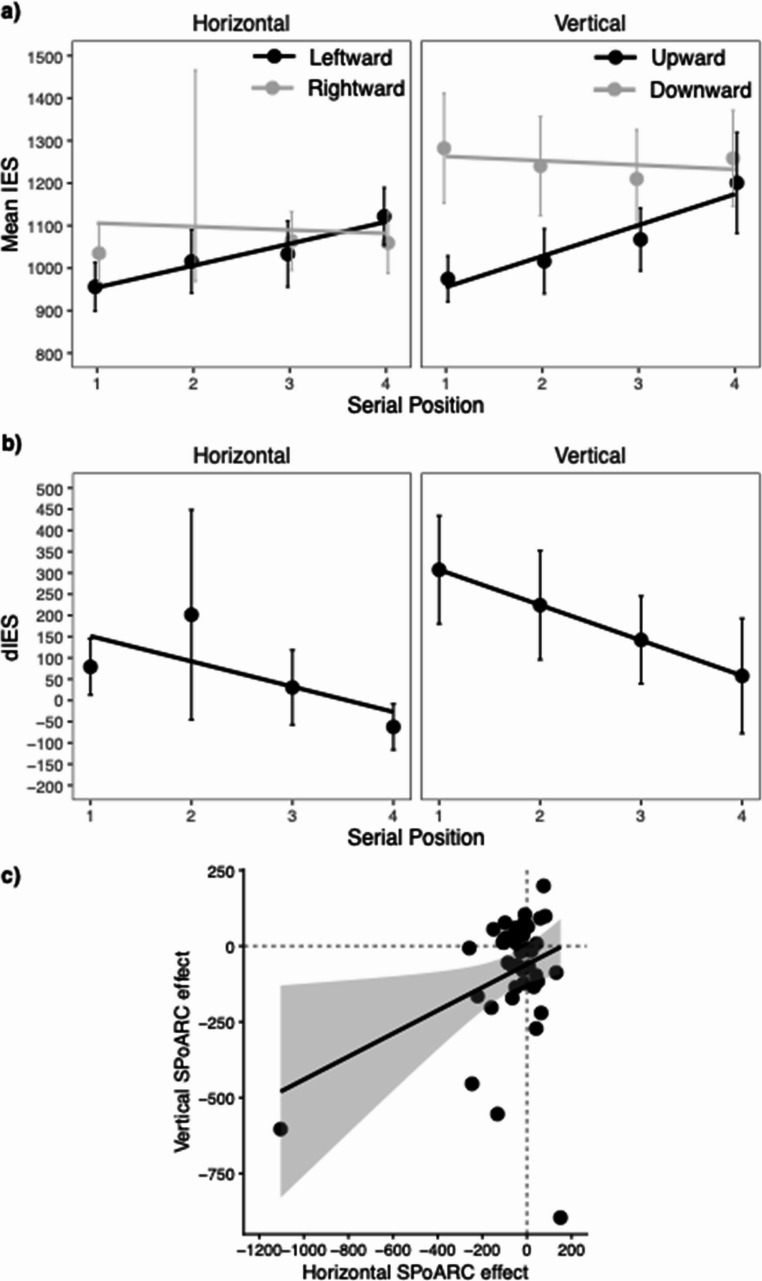
Mean IES, mean dIES and correlation between horizontal and vertical SPoARC effect in Experiment 2. The upper panel (**a**) shows mean IES for each response direction and serial position. The middle panel (**b**) shows mean dIES (horizontal: right minus left; vertical: down minus up) for each serial position. Error bars depict +/- 1 SEM. The lower panel (**c**) shows the correlation between the horizontal and vertical SPoARC effect (individual regression slopes of dIES as a function of serial position). The solid line shows the linear fit (with +/- 95 CI). The dashed lines mark the boundaries for positive and negative SPoARC effects, with dots in the lower left quadrant reflecting the concurrent presence of early-leftward/and early-upward associations

The analysis revealed a significant effect of response direction, *F*(1, 638) = 9.22, *p* =.002, with better performance (lower IES) for leftward/upward responses (*M* = 1048, *SEM* = 63) compared to rightward/downward responses (*M* = 1170, *SEM* = 101), and a significant effect of orientation, *F*(1, 638) = 5.37, *p* =.021, with better performance for the horizontal (*M* = 1062, *SEM* = 72) compared to the vertical (*M* = 1156, *SEM* = 78) spatial orientation. Most importantly, there was a significant interaction between serial position and response direction, indicating a SPoARC effect across orientations, *F*(1, 638) = 3.91, *p* =.049. Τhe SPoARC effect did not interact with orientation (*F* < 1). Analysis of the individual regression coefficients (betas) for the dIES as a function of serial position confirmed a significant horizontal SPoARC effect, *t*(42) = −2.09, *p* =.042, Cohen’s *d* = −0.32, although the associated BF_10_ of 1.19 suggests only weak evidence supporting the horizontal SPoARC effect. The mean slope was − 59.51 ms (*SEM* = 28). The horizontal SPoARC effect was also confirmed by non-parametric Wilcoxon sign rank test (*p* =.014). The analysis also confirmed that there was a significant vertical SPoARC effect, *t*(42) = −2.63, *p* =.011, Cohen’s *d* = −0.40. The mean slope was − 83.12 ms (*SEM* = 32). The BF_10_ of 3.41 indicates moderate support for the presence of a vertical SPoARC effect. The vertical SPoARC effect was also confirmed by non-parametric Wilcoxon sign rank test (*p* =.034). Finally, while parametric testing revealed a significant correlation between the horizontal and vertical SPoARC slopes (*r* =.34, *p* =.025, BF_10_ = 3.16), this correlation was no longer significant with non-parametric correlation testing, Spearman’s rho = 0.12, *p* =.450, suggesting that the former correlation is biased by the extreme values. Please refer to Appendix B for the associated Table B2 and Fig. B2.

#### Comparison between experiments

First, we checked the behavior of SPoARC correlations (on slopes) across orientations (horizontal vs. vertical) and measures (RT vs. ER), and descriptively compared this between experiments (Table 6). Notable observations are (a) that the correlation between the horizontal SPoARC effect on RTs and the vertical SPoARC effect on ERs is not significant in either of the experiments, (b) that none of the correlations is significant for Experiment 2, while several reliable correlations are observed for Experiment 1, and (c) that the correlation coefficients are generally larger in Experiment 1 than Experiment 2. Overall, then, we believe that SPoARC correlations behave differently between experiments, which is elaborated on below.

**Table 6:** Correlations for all dRT and dER slopes for both experiments

Pairwise correlation	Experiment 1			Experiment 2		
	*r*	*p*	BF_10_	*r*	*p*	BF_10_
RT Horizontal – RT Vertical	0.42	0.016	4.69	< 0.01	0.981	0.34
RT Horizontal – ER Horizontal	0.37	0.034	2.67	0.15	0.323	0.53
RT Horizontal – ER Vertical	0.15	0.406	0.52	0.11	0.498	0.42
RT Vertical – ER Horizontal	0.24	0.173	0.85	− 0.08	0.607	0.38
RT Vertical – ER Vertical	− 0.18	0.320	0.59	− 0.19	0.215	0.68
ER Horizontal – ER Vertical	0.52	0.002	23.79	0.09	0.573	0.39

Second, omnibus analyses were performed in order to directly compare the SPoARC effect between experiments. We opted to perform these analyses on slopes (rather than the full-blown mixed-effects models) for the purpose of brevity. As becomes clear from Table 7, the SPoARC effect as indexed by slopes (across orientation and measure) was significantly reduced in Experiment 2 (as compared to Experiment 1) for the horizontal SPoARC effect on ERs and the vertical SPoARC effect on RTs, while there was a trend towards reduction for the vertical SPoARC effect on ERs.

**Table 7:** Effect of experiment (1, 2) on the spoarc effect

RT Horizontal	T	*p*	Cohen’s d	BF_10_
	−0.06	0.952	−0.01	0.24
RT Vertical	−2.59	0.012	−0.60	4.03
ER Horizontal	−3.85	< 0.001	−0.89	99.97
ER Vertical	−1.69	0.094	−0.39	0.82

*df* = 74 for all tests. Results are based of independent sample *t*-tests with the grouping variable Experiment (1, 2) on the SPoARC slopes. BF_10_ = Bayes Factor

## Discussion

The multidimensional scenario provided the best fit to the outcomes of Experiment 1. One purpose of Experiment 2 was to put this scenario further to the test. Experiment 2 altered the trial-design of Experiment 1 by adding a cue that informed participants about the saccade response boxes for vowel-consonant classification just before a target letter was presented. As expected, this addition increased the cognitive demands of the task (i.e. enhanced response latencies and error rates) as compared to Experiment 1, but what happened with the theoretically more relevant observations concerning the SPoARC effects? Was our prediction correct that this adjustment would reduce the tendency for multidimensional coding due to (a) removing the potential cue in the design (i.e. a fixed mapping that connects left-top and right-bottom to each other) for diagonal coding, and/or (b) reducing mental resources that would presumably be needed for keeping active multiple order representations at the same time?

We observed a reliable horizontal SPoARC effect on RTs but not (or only by trend) on ERs, while a vertical SPoARC effect was only observed in ERs (IES analyses confirmed the presence of both SPoARC effects overall). Moreover, these effects did not correlate with each other. Hence, on the one hand, there was a clear impact of the design as compared to Experiment 1, where we observed reliable and correlated SPoARC effects across all orientations and measures. This indicates that the adjustments made to the design had a significant impact on the outcomes, as also shown by the between-experiment analyses above (and discussed below). On the other hand, a significant SPoARC effect was still observed for both horizontal (on RTs) and vertical (on ERs) trials (cf. also the IES analyses), so did the adjustments also negatively affect the tendency for multidimensional coding (as was predicted)?

First, we believe that Experiment 2 outcomes argue against the exploitation of a diagonally oriented representation. If a single, diagonally oriented representation was mentally scanned to determine the required response, and if these internal spatial reorientations are responsible for the horizontal SPoARC effect in Experiment 2, then we would expect a vertical SPoARC effect on RTs to accompany its horizontal counterpart. Specifically, the scanning of a diagonally oriented order representation drives spatial reorientations (i.e. moving from one item in the sequence to another) in both the horizontal and vertical planes. Moreover, a diagonal line would strongly predict the horizontal and vertical SPoARC effects to be correlated, as they derive from the same scanning process, and such correlation was not observed (but, such correlation between horizontal SPoARC on RT and vertical SPoARC on ER effects was neither observed in Experiment 1; see Table 6). So, within the multidimensional scenario, the findings of Experiment 2 no longer fit the strategy of diagonal coding.

Second, what about the strategy of simultaneously exploiting a horizontally and vertically oriented order representation (multiple mental lines being maintained within an individual)? Did the added manipulation in Experiment 2 reduce the tendency to engage in this strategy, as compared to Experiment 1? Here the data are less clear. In Experiment 2 we still observed a significant SPoARC effect for both the horizontal (on RTs) and the vertical (on ERs) trials, which does not fit the notion that individuals generally fell back to a unidimensional-homogeneous or no-spatialization scenario. This finding in itself aligns with either the unidimensional-heterogeneous scenario, or the strategy of exploiting multiple mental lines from the multidimensional scenario. Whereas neither of these two scenarios is fully supported by the (absent) SPoARC correlations, it is notable that the correlation coefficient between the horizontal (on RTs) and vertical (on ERs) SPoARC effects is positive in absolute sense (*r* =.11), which does not fit the unidimensional-heterogeneous scenario (which would predict a negative correlation coefficient). So, building on Table 1 predictions alone, the findings of Experiment 2 may still be best interpreted as indicating a strategy of holding active multiple mental lines simultaneously.

Nevertheless, the added manipulation in Experiment 2 had a notable impact on behavior as compared to Experiment 1 – both in terms of overall task difficulty (e.g., increased RTs and ERs) and in terms of general SPoARC patterns. Specifically, the SPoARC correlations behaved differently between experiments (being present in Experiment 1 but not Experiment 2), and the SPoARC effects were less pronounced in Experiment 2 as compared to Experiment 1 (cf. omnibus analyses above). So, whereas the findings of Experiment 2 do not fit the notion that multidimensional coding was fully abolished, we believe that – if still present – this tendency was at least reduced as compared to Experiment 1. Overall, the findings of Experiment 2 would fit best the conclusion that the new manipulation demotivated multidimensional coding for some (but not all) participants, and these participants fell back to unidimensional coding with diversity in the precise orientation that was adopted (i.e. unidimensional-heterogeneous scenario). Across the group, this would approximately fit the findings of uncorrelated horizontal and vertical SPoARC effects.

Overall, we conclude that the strength of the evidence for multidimensional coding is much weaker in Experiment 2 than was the case for Experiment 1 – in line with what we predicted. This fits the notion that the tendency for multidimensional coding in Experiment 1 was driven either (a) by diagonally oriented mental lines, the exploitation of which was abolished in Experiment 2 due to the breakdown of a strong left-top and right-bottom coupling in the response rules; or (b) by the simultaneous exploitation of a horizontal and vertical line, which was reduced in Experiment 2 due to increased task load.

### General discussion

The current study aimed to explore the possibility of multidimensional coding on the mental whiteboard, in order to test its promise as a two-dimensional workspace that can be functionally deployed in tasks building on verbal working memory. Previous work already demonstrated the flexible use of the mental whiteboard in response to specific task features (e.g. Guida et al., 2020; Hartmann et al., 2021), in line with a goal-directed, context-specific mechanism. But in the designs of these previous studies, people could suffice with flexible coding on single, unidimensional mental lines. Here we attempted to implement a design that probes the full use of the 2D mental whiteboard, to explore if people are capable of such. Specifically, Hartmann et al. (2021) showed that a person can use either a horizontally or a vertically oriented representation in function of the orientation of saccade response boxes in the task. As a follow-up, here we changed the task demands by varying the response box orientation from trial to trial – rather than across different sessions such as in the study by Hartmann et al. (2021). In Experiment 1, we observed that in such a design, both horizontal and vertical SPoARC effects are present – and that they positively correlate, in line with the notion that they share their underlying mechanism. This novel empirical finding is here interpreted as the first support for multidimensional coding on the mental whiteboard, going beyond the use of a single, unidimensional mental line.

Though the findings of Experiment 2 are more difficult to interpret with high confidence (see below for elaboration), they do suggest that changes to the overall task context (in this case, a cue that needed to be processed to determine the precise classification rules for the current trial) can partially breakdown the tendency for multidimensional coding – in line with spatial coding for serial order being a context-appropriate, goal-directed mechanism (cf. mental whiteboard hypothesis). With the overall task context no longer providing incentives (i.e. the classification rules no longer probing diagonal coding) and/or leaving sufficient mental resources (i.e. increased task difficulty preventing the exploitation of multiple mental lines) for multidimensional coding, we come to the following conclusion for the group-level findings of Experiments 1 and 2: Whereas participants exploiting multidimensional coding dominated the data pattern in Experiment 1, in Experiment 2 there was a more balanced combination of multidimensional coding and unidimensional-heterogeneous coding (see above for elaboration).

Finally, the replication of a top-to-bottom orientation for vertically outlined order representations (cf. Hartmann et al., 2021) in especially Experiment 1 further goes against existing accounts that could explain away spatial effects in serial order tasks as epiphenomenal (i.e. polarity coding, metaphor theory, and indirect spatial-numerical association logic). All these alternative accounts would – under default conditions – predict a bottom-to-top orientation. Indeed, these epiphenomenal accounts would not be readily able to explain why the mere addition of response mapping uncertainty would impact the findings from Experiment 1 to Experiment 2. Mental whiteboard logic better fits such task set effects, as spatial codes are here intrinsic part of goal-directed task setting – which implies task-customization.

### Cognitive control over the building and the use of serial order representations

The hypothesis that (spontaneous) spatial coding is a functional tool of verbal working memory that is put to use in a goal-directed manner when confronted with serial order demands (cf. mental whiteboard analysis), invites an integration of serial order processes into the more general cognitive control theories. For example, the flexibility in operating on the mental whiteboard that has been shown in previous work (e.g. Guida et al., 2020; Hartmann et al., 2021) as well as the current study, can be framed in terms of the Dual Mechanisms of Control framework (Braver, 2012). This framework postulates that humans regulate their thoughts and actions through two distinct modes of cognitive control: a proactive mode for preparatory or anticipatory regulation before a demanding cognitive task or stimulus appears, and a reactive mode that serves to quickly adjust when ongoing task demands are confronted with a suboptimal mental configuration. The flexible calibration of serial order coding on the mental whiteboard to specific task demands, is a type of proactive control, as it occurs in preparation (i.e. encoding phase) of the trials in which retrieval of serial order is required (i.e. the go-nogo classification task). Hence, the mental whiteboard is proactively installed when facing serial order encoding demands, and the orientation of items on it are proactively aligned with task context.

Interestingly, the multidimensional scenario outlined above, describes a possible strategy that would also include reactive control: If participants proactively encode and maintain not only one (as indicated by previous work) but multiple distinct spatially defined order representations – a horizontally oriented representation to use for horizontal trials, and a vertically oriented representation to use on vertical trials – then reactive control would be required on each trial to select the currently operational serial order representation *on the fly* depending on current trial context. As such, it would still be very interesting to test the two strategies against each other that above were categorized together under the multidimensional coding scenario: Multidimensional coding was assumed to imply either (a) the *proactive* use of a single, diagonally oriented mental line, or (b) the use of both a horizontally and a vertically oriented order representation. Only the latter implies reactive control.

If indeed multiple mental lines are maintained and switched across trials within the same block, selecting at each trial the mental line which orientation fits best the trial context (i.e. the orientation of response boxes), then switch costs may be predicted to occur when switching between trials with different orientations. Such switch costs are a robust observation in switching between other types of task representations (i.e. task sets; Egner, 2023), and repeating a specific orientation across trials may equally facilitate the agent’s mental work. Here we performed some additional, exploratory analyses on the current data in search of such *orientation switch costs* (Appendix C) – focusing especially on nogo-go trial successions. Specifically, even though orientation switch costs may appear between successive go-trials, repeating or switching between order representations in go-go trial successions may entail various other task-related processes as well that are repeated or switched, as the stimulus-response task is executed across both trials. Therefore, we believe that a more reliable assessment of orientation switch costs can be obtained specifically for go-trials that are preceded by a nogo-trial, as the nogo-trial still requires a participant to scan the sequence in memory without producing a subsequent response – thus minimizing switch costs that may be related to switching between actually implemented stimulus-response rules. Overall, orientation switch costs were not observed in Experiment 1 (and neither in Experiment 2). At this point, then, we argue that Experiment 1 findings best fit a scenario in which people adopt a diagonally oriented order representation that can fit well enough both trials with horizontally outlined response boxes and trials with vertically outlined response boxes. In Experiment 2, such diagonal coding was partially abolished.

Overall, the mental whiteboard hypothesis provides a concrete implementation of attention-based mechanisms for serial order (e.g. Camos, 2015) on top of the classical phonological loop. It postulates a critical role for internal spatial attention in the encoding, maintenance, and retrieval of items from serial order working memory. In the current paper we aimed to make a closer link to the general literature on attentional and cognitive control, reporting on a novel finding of multidimensional coding on the mental whiteboard – whenever the tasks probes and/or allows for it. We hope that future studies will further pursuit the theoretical and methodological cross-fertilization between the domains of serial order working memory and cognitive control more broadly.

## Figures and Tables

**Fig. A1:**
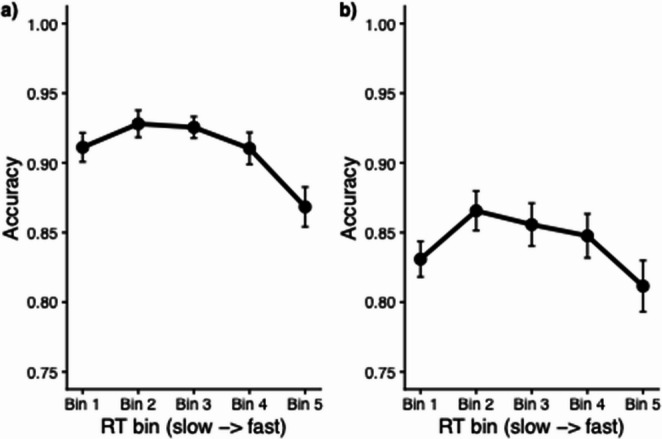
Conditional accuracy function for Experiment 1 (panel **a**) and Experiment 2 (panel **b**)

## Data Availability

We report how we determined our sample size, all data exclusions, all manipulations, and all measures in the study. All data, analysis code, and research materials are available at [https://osf.io/6krq9/](https:/osf.io/6krq9). We have complied with APA ethical standards in the treatment of participants and their data. This study’s design and its analysis were not pre-registered.

## Appendix A: Speed-accuracy trade-offs

To assess potential speed–accuracy trade-offs, we examined accuracy as a function of response time using conditional accuracy functions (CAFs). For Experiment 1 and 2 separately, response times were first divided into five quantile-based bins separately for each participant and each combination of orientation, serial order, and response direction, ensuring equal representation of all factor combinations within each bin. Accuracy was then averaged within each participant and bin. Visual inspection of the CAFs revealed a characteristic pattern for both experiments (see Fig. A1): Accuracy decreased monotonically across bins 2–5, indicating that faster responses were associated with lower accuracy. In addition, accuracy was reduced in the slowest response-time bin. Fast errors (fastest bin) are commonly interpreted as reflecting impulsive responding or insufficient evidence accumulation, whereas slow errors (slowest bin) are often attributed to attentional lapses or temporary disengagement from the task (e.g., mind-wandering). Overall, the observed CAFs exhibit the typical pattern associated with speed–accuracy trade-offs in both Experiment 1 and 2.

## Appendix B: Tables and figures complementing the IES analyses

**Table B1:** Summary of IES analyses for Experiment 1

	$F$	$p$
Response direction (RD)	10.34	.001
Serial position (SP)	5.27	.022
Orientation (O)	2.21	.138
RD x SP (SPoARC effect)	61.17	<.001
RD x O	9.65	.002
SP x O	< 0.01	.999
RD x SP x O	0.23	.630

**Table B2:** Summary of IES analyses for Experiment 2

	$F$	$p$
Response direction (RD)	9.22	.002
Serial position (SP)	2.17	.141
Orientation (O)	5.37	.021
RD x SP (SPoARC effect)	3.91	.049
RD x O	2.24	.135
SP x O	0.07	.789
RD x SP x O	0.11	.744

## Appendix C: Orientation switch costs

The goal of these analyses was to zoom in on the precise mechanism of the multidimensional coding scenario, for which Experiment 1 and 2 provided support (see main text of the paper). Specifically, we aimed to distinguish between (i) diagonal coding of order on the mental whiteboard (a representation would be of use for both vertical and horizontal trials), and (ii) the active maintenance of independent horizontally and vertically oriented order representations. The latter (but not the former) strategy would possibly predict switch costs to occur when switching between the two orientations. Such switch costs are a robust observation in switching between other types of task representations (i.e. task sets; Egner, 2023), and similar mental reconfiguration may be required when switching from an active horizontal to an active vertical mental line. As noted in main text, we believe that the most reliable assessment of orientation switch costs can be obtained specifically for go-trials that are preceded by a nogo-trial. We specifically focused on RTs since the SPoARC effects on RTs are the standard observation taken to indicate operations on the mental whiteboard. Finally, the main interest for switch costs concerns the data from Experiment 1 (as there the horizontal and vertical SPoARC effects were most reliably observed), but the same analyses are here reported for Experiment 2 for the sake of completeness.

Thus, we assessed whether switching the response box orientation (from horizontal to vertical or vice versa) from one trial to the next led to costs in response latencies for nogo-go trial sequences (cf. General Discussion section of the paper). To address this question, we created the variable orientation switch (switch, no switch). The linear mixed-effects model described in the main article was repeated with the additional fixed effects orientation switch, as well as the interaction between orientation switch and orientation to capture a possible asymmetry in switch costs from one orientation to the other. Only pairs of correct trials were included to avoid post-error slowing effects.

### Experiment 1

As noted in the introduction, the presumably cleanest assessment of orientation switch costs in the current study involves nogo-go trial sequences. A total of 3892 trials were suitable for such an analysis. All of these trials were correct go-responses following a correct nogo trial. If the response alignment of the previous trial was in the same spatial orientation, the trial was classified as no-switch (*n* = 1959), otherwise as switch (*n* = 1933). For each participant, mean latencies were computed for each serial position, response direction, orientation and orientation switch (switch, no switch) and the linear mixed-effects model analysis of RTs was repeated with the additional fixed effect orientation switch and the interaction between orientation switch and orientation.

The analysis replicated the significant effect of serial position, *F*(1, 31.70) = 20.13, *p* <.001, a significant interaction between serial position and response direction (SPoARC effect), *F*(1, 927.63) = 37.93, *p* <.001, and also a significant interaction between orientation and response direction, *F*(1, 925.05) = 7.68, *p* <.006 (cf. main analysis). All other effects were not significant (*p*’s > 0.098). Importantly, there was clearly no effect of orientation switch, *F*(1, 926.17) = 0.02, *p* =.882. RTs were nearly identical for switch (*M* = 692, *SEM* = 9) and no-switch trials (*M* = 693, *SEM* = 9). There was also no interaction between orientation switch and orientation, *F*(1, 924.22) = 0.72, *p* =.397.

### Experiment 2

As in Experiment 1, we analyzed switch costs in nogo-go trial sequences. A total of 4627 trials were suitable for such an analysis. All of these trials were correct go-responses following a correct nogo trial. If the response alignment of the previous trial was in the same spatial orientation, the trial was classified as no-switch (*n* = 2352), otherwise as switch (*n* = 2275). The analysis replicated the significant effect of serial position, *F*(1, 42.12) = 24.12, *p* <.001, a significant interaction between response direction and orientation, *F*(1, 1193.86) = 5.93, *p* =.015, as well as the three-way interaction between serial position, response direction and orientation, *F*(1, 1194.83) = 4.01, *p* =.045. All other effects were not significant (*p*’s > 0.135). Importantly, there was again no effect of orientation switch, *F*(1, 1193.59) = 1.01, *p* =.314. RTs were similar for switch (*M* = 890, *SEM* = 12) and no-switch trials (*M* = 893, *SEM* = 12). There was no interaction between orientation switch and orientation, *F*(1, 1193.92) = 1.86, *p* =.173.

## Footnotes

1 Spontaneous‘ in the sense that the non-spatial format in which the verbal item sequence is presented to the participant (e.g., auditory presentation of items, or visual presentation of items at the center of the screen) does not provide external cues or demands for spatial coding.

2 A sequence of verbal items that was presented in a non-spatial format during encoding, for example by presenting all items serially at the center of the screen.

3 Zhou et al. (2019) showed a bottom-to-top mapping in Chinese participants, which contrasts with the Mental Whiteboard hypothesis at first sight. However, as noted by the authors, the use of the vertical orientation in reading/writing is used infrequently in everyday life in Chinese culture, and we argue that in such case the orientation may be shaped by other directional drivers such as metaphors.

4 We acknowledge the existence of different kinds of spatial-numerical associations, each with its specific underlying cognitive mechanisms (see Cipora et al., 2020). Additionally, it is known that spatial-numerical associations can be flexibly remapped via task context or instructions in both horizontal (e.g. Bächtold et al., 1998) and vertical axes (e.g. Hartmann et al., 2014; Felisatti et al., 2024). For example, Hartmann et al. (2014) showed reversed vertical SNARC effects depending on which set of effectors was used. However, here we base our predictions on a large set of studies (e.g. Aleotti et al., 2020; Hartmann et al., 2014; Greenacre et al., 2022; Ito & Hatta, 2004) showing that – in typically used classification tasks like parity judgement or magnitude comparison – numbers are associated to vertical space from bottom (small numbers) to top (large numbers). This also holds for eye movement responses (Schwarz & Keus, 2004).

5 This cuing was added in order to prevent participants from the need to make eye movements towards the actual response boxes to identify response directions while the target letter is already on the screen (thus ensuring that eye movements are made solely for classification responses).
